# Longitudinal Evolution of the Pseudomonas-Derived Cephalosporinase (PDC) Structure and Activity in a Cystic Fibrosis Patient Treated with β-Lactams

**DOI:** 10.1128/mbio.01663-22

**Published:** 2022-09-08

**Authors:** Claudia A. Colque, Andrea G. albarracín Orio, Pablo E. Tomatis, Gina Dotta, Diego M. Moreno, Laura G. Hedemann, Rachel A. Hickman, Lea M. Sommer, Sofía Feliziani, Alejandro J. Moyano, Robert A. Bonomo, Helle K. Johansen, Søren Molin, Alejandro J. Vila, Andrea M. Smania

**Affiliations:** a Universidad Nacional de Córdoba, Facultad de Ciencias Químicas, Departamento de Química Biológica Ranwel Caputto, Córdoba, Argentina; b CONICET, Universidad Nacional de Córdoba, Centro de Investigaciones en Química Biológica de Córdoba (CIQUIBIC), Córdoba, Argentina; c Instituto de Biología Molecular y Celular de Rosario (IBR), CONICET, Universidad Nacional de Rosario, Rosario, Argentina; d Facultad de Ciencias Bioquímicas y Farmacéuticas, Universidad Nacional de Rosario, Rosario, Argentina; e IRNASUS, Universidad Católica de Córdoba, CONICET, Facultad de Ciencias Agropecuarias, Córdoba, Argentina; f IQUIR, Instituto de Química de Rosario, CONICET, Universidad Nacional de Rosario, Rosario, Argentina; g Department of Clinical Microbiology, Rigshospitalet, Copenhagen, Denmark; h Novo Nordisk Foundation Centre for Biosustainability, Technical University of Denmark, Lyngby, Denmark; i Departments of Molecular Biology and Microbiology, Medicine, Biochemistry, Pharmacology, and Proteomics and Bioinformatics, Case Western Reserve University, Cleveland, Ohio, USA; j Senior Clinical Scientist Investigator, Louis Stokes Cleveland Department of Veterans Affairs, Cleveland, Ohio, USA; k Department of Clinical Medicine, University of Copenhagen, Copenhagen, Denmark; McMaster University

**Keywords:** *Pseudomonas aeruginosa*, cystic fibrosis, β-lactamase evolution, ceftolozane resistance, hypermutability

## Abstract

Traditional studies on the evolution of antibiotic resistance development use approaches that can range from laboratory-based experimental studies, to epidemiological surveillance, to sequencing of clinical isolates. However, evolutionary trajectories also depend on the environment in which selection takes place, compelling the need to more deeply investigate the impact of environmental complexities and their dynamics over time. Herein, we explored the within-patient adaptive long-term evolution of a Pseudomonas aeruginosa hypermutator lineage in the airways of a cystic fibrosis (CF) patient by performing a chronological tracking of mutations that occurred in different subpopulations; our results demonstrated parallel evolution events in the chromosomally encoded class C β-lactamase (*bla*_PDC_). These multiple mutations within *bla*_PDC_ shaped diverse coexisting alleles, whose frequency dynamics responded to the changing antibiotic selective pressures for more than 26 years of chronic infection. Importantly, the combination of the cumulative mutations in *bla*_PDC_ provided structural and functional protein changes that resulted in a continuous enhancement of its catalytic efficiency and high level of cephalosporin resistance. This evolution was linked to the persistent treatment with ceftazidime, which we demonstrated selected for variants with robust catalytic activity against this expanded-spectrum cephalosporin. A “gain of function” of collateral resistance toward ceftolozane, a more recently introduced cephalosporin that was not prescribed to this patient, was also observed, and the biochemical basis of this cross-resistance phenomenon was elucidated. This work unveils the evolutionary trajectories paved by bacteria toward a multidrug-resistant phenotype, driven by decades of antibiotic treatment in the natural CF environmental setting.

## INTRODUCTION

Our current knowledge regarding the evolution of bacterial antibiotic resistance derives from clinical, microbiological, and biochemical studies that are performed under controlled conditions (1–7). Collectively, we have learned that the emergence and evolution of antibiotic resistance, one of the greatest challenges to our civilization, are a far more complex phenomena, and only a few studies offer insights into “real world” scenarios that can adequately or completely explain the evolutionary trajectories that shape existing resistance phenotypes (8–14).

Chronic infections by Pseudomonas aeruginosa are the main causes of morbidity and mortality in patients with cystic fibrosis (CF). Treating these long-term airway infections is extremely challenging since P. aeruginosa displays an intrinsic resistance to many antibiotics, as well as an unwelcome capacity to develop and evolve resistance to newly introduced antibiotics. Acquired antibiotic resistance in CF-associated isolates of P. aeruginosa occurs mainly through the accumulation of multiple mutations that alter the expression and/or function of different chromosomal genes (15, 16). Furthermore, P. aeruginosa hypermutator strains are frequently isolated from CF patients, thus increasing the pace of antibiotic resistance development (17–21) and the repertoire of adaptive phenotypes (22–27). Nevertheless, CF persistent infections offer unique opportunities to study antibiotic resistance evolution due to (i) the long-term antibiotic treatments to which patients are subjected during their entire lives, (ii) the most often clonal P. aeruginosa lineages that persist in the lungs of individual patients with accessible and regular sampling, and (iii) the growing availability of new and improved tools for genomics, transcriptomics, and proteomics analysis. Thus, *de novo* evolution of antibiotic resistance in individual patients can be monitored, providing time-resolved maps of the evolutionary trajectories of the infecting bacteria (28).

A varied repertoire of antipseudomonal antibiotics is used for treatment of CF respiratory tract infections, including aminoglycosides, quinolones, and β-lactams. In response, P. aeruginosa displays a wide arsenal of resistance mechanisms, such as reduced outer membrane permeability, upregulation of multiple broad-spectrum drug efflux pumps, antimicrobial-modifying enzymes, and target site changes (29). The main resistance mechanism against β-lactams is the expression of the chromosomally encoded class C β-lactamase PDC (*Pseudomonas-*derived cephalosporinase). Constitutive overexpression of this intrinsic *ampC* gene (here, *bla*_PDC_) results from mutations affecting regulatory genes of the peptidoglycan recycling process linked to bacterial cell wall assembly (30–34). β-Lactam resistance has also been associated with structural modifications of PDC (35–41), as evidenced by the report of >400 PDC variants (42). This impressive number of allelic variants accounts for a highly polymorphic enzyme with a great capacity of tolerating amino acid substitutions, insertions, and deletions (42). Recent studies have shown that clinical resistance to β-lactams is primarily based on specific changes in conserved motifs of PDC, which lead to conformational rearrangements enhancing the catalytic efficiency of the enzyme (38, 43–46). These findings highlight the need to identify all new variants that provide more robust resistant phenotypes.

A whole-genome sequencing (WGS) study of hypermutator populations of P. aeruginosa during long-term chronic airway infections in a single patient (27) revealed mutations in 42 genes (from a total of 77) in the β-lactam resistome (47). Specifically, the *bla*_PDC_ gene was targeted by multiple independent mutational events in a process accelerated by hypermutability leading to a wide diversity of coexisting *bla*_PDC_ alleles and high levels of β-lactam resistance (47). However, the presence of mutations in several genes and the increased expression of PDC compared to that in isolates preceding the antibiotic treatment do not permit a direct assessment of the impact of the allelic variability of PDC in resistance. Therefore, the dissection of the impact of specific mutations in the PDC gene is essential to trace the evolution of this enzyme and consequently to guide future therapies targeting PDC.

Herein, we unravel the mutational pathways and biochemical mechanisms involved in the evolution of different PDC variants by examining isolates from more than 25 years of treatment of CF chronic infection in a single patient. We show how the combination of substitutions in important amino acid residues in PDC shapes the architecture of the enzyme active site. This adaptive scenario led to the selection of distinct β-lactamase variants, which conferred resistance to a broad range of β-lactam antibiotics, even to novel combinations of these drugs that were not prescribed to this patient, such as ceftolozane-tazobactam. We also identify the molecular features that elicited the selection of collateral resistance driven by the use of broad-spectrum cephalosporins and how this resistance was potentiated by hypermutability in P. aeruginosa. By modeling a core of three substitutions preserved in the prevailing variants, we hypothesize that specific interactions between ceftazidime and PDC in these variants enhance the ability of this enzyme to bind and cleave ceftolozane. This work details the trajectory undertaken on the path toward a multidrug-resistant phenotype, even against untested drugs, and provides the molecular mechanisms leading to this “collateral damage” upon years of antibiotic treatment in the attempt to eradicate this versatile pathogen.

## RESULTS

### Long-term evolution of P. aeruginosa hypermutator populations leads to the selection of novel *bla*_PDC_ allelic variants with enhanced cephalosporin resistance.

**FIG 1:**
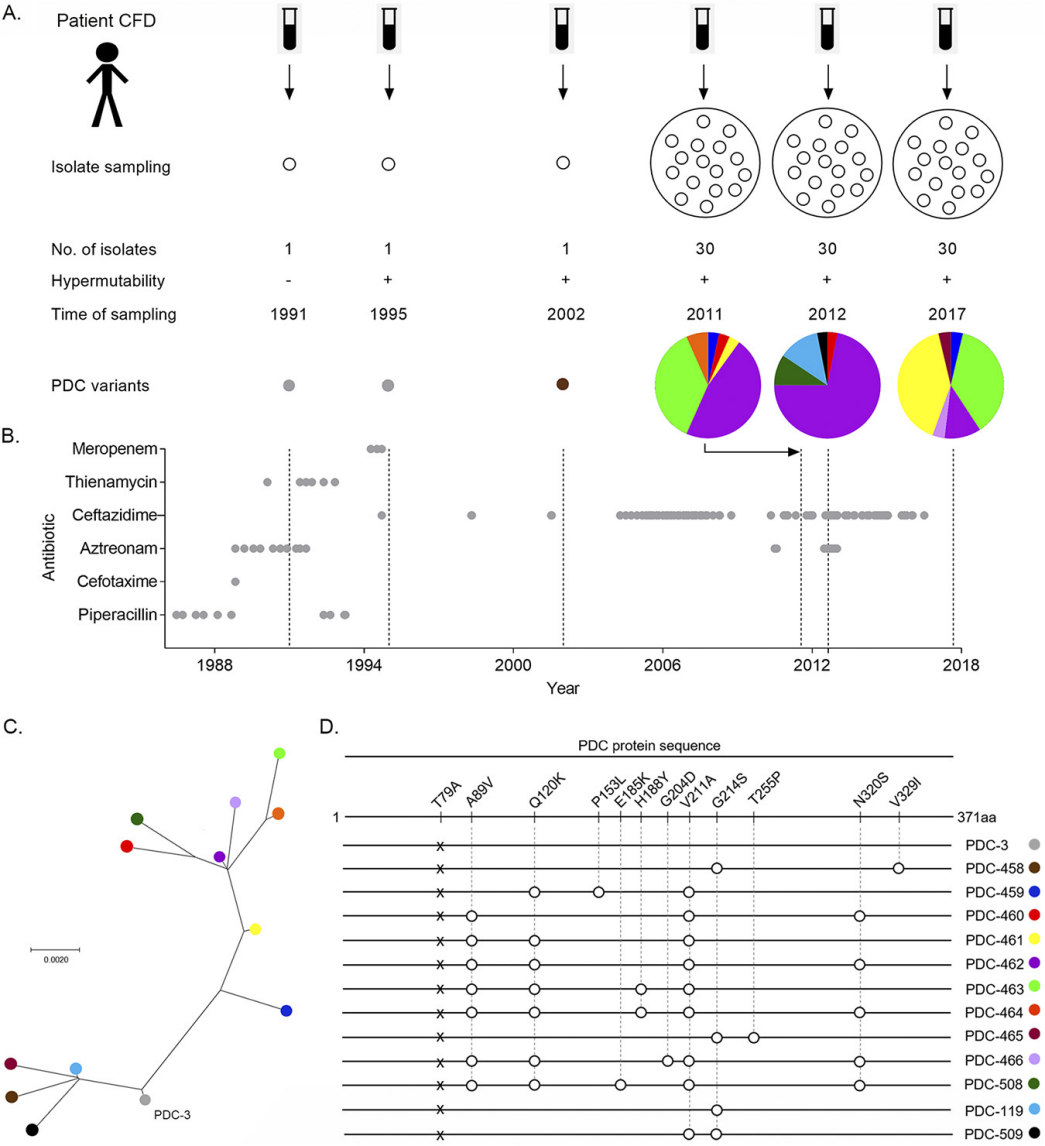
Evolution of β-lactamase PDC variants in CFD isolates. (A) Overview of P. aeruginosa isolates collected from patient CFD throughout the 26-year study. Isolate sampling from sputum samples covered a period from 1991 to 2017. The collection included single isolates from 1991, 1995, and 2002, as well as populations of 30 isolates from single sputum samples in 2011, 2012, and 2017. The plus and minus symbols indicate the hypermutability state of the P. aeruginosa strains. PDC colored variants and pie charts representing the percentage of each PDC variant in the 3 populations are shown. (B) β-Lactam antibiotic treatment received by the CFD patient. Antibiotics used in chemotherapy are listed on the *y* axis. Treatment with this group started from 1986 and lasted until the end of 2016. Gray circles indicate the start and end of an antibiotic dose. (C) Phylogenetic reconstruction of PDC variants. The PDC-3 sequence was used for rooting the tree (see the supplemental material for details). Circle colors represent different types of PDC variants found in CFD isolates. (D) Schematic representation of the β-lactamase PDC protein sequence strain PAO1 and of the 13 PDC variants that emerged during the 26 years of evolution with their amino acid variations with respect to PAO1. Numbering of amino acids refers to the mature protein of PAO1 strain, after cleavage of the 26 N-terminal amino acid residues from the signal peptide, according to the PDC-wide structural position system (SANC numbering) (59). PDC-3 differs from PDC-1 from PAO1 by the T79A mutation, which does not affect resistance or the substrate specificity of the lactamase (37, 48). Early isolates from 1991 and 1995 harbor PDC-3. The isolate from 2002 harbored the PDC-458 variant. The set of 30 isolates evaluated in 2011, 2012, and 2017 contained different coexisting variants: 2011, PDC-459 to PDC-464 variants, 2012, PDC-460, -462, -508, and -119; and 2017, PDC-459, -461, -462, -463, -465, and -466.

The complete genomes of 14 isolates from a hypermutator P. aeruginosa lineage spanning 20 years of patient infection history (CFD patient) were recently reported (27). The clonal collection included a nonmutator ancestor from 1991, two hypermutator isolates from 1995 and 2002, and 11 isolates taken from the same sputum sample in 2011, all of them harboring the same *mutS* mutation (27) (Fig. 1A). Within-patient genome comparisons revealed a vast accumulation of mutations that shape an extensively diversified population composed of different sublineages, which coexisted from the beginning of the chronic infection. Within the β-lactam resistome, the gene encoding the β-lactamase PDC (*bla*_PDC_) was among the most frequently altered by mutations across different isolates, suggesting that *bla*_PDC_ was subjected to strong selective pressure (47) (see Fig. S1 in the supplemental material; https://www.researchgate.net/publication/361368769_Supplemental_Material_for_Longitudinal_evolution_of_the_Pseudomonas_Derived_Cephalosporinase_PDC_structure_and_3_activity_in_a_Cystic_Fibrosis_patient_treated_with_b-lactams). In fact, during the course of chronic airway infection, the patient received prolonged antibiotic treatment with β-lactam antibiotics (Fig. 1B). The patient initially received short courses of variable duration of cefotaxime, ceftazidime, piperacillin, aztreonam, and two carbapenems (thienamycin and meropenem); then he was intermittently treated with ceftazidime from 2004 until the end of 2016 (Fig. 1B).

10.1128/mbio.01663-22.2 FIG S1 Mutations potentially affecting protein function of 42 out of the 77 genes related to β-lactam were analyzed. A heat map of the number of mutations accumulated per gene in each genome is shown. Isolates were grouped based on hierarchical clustering using the pheatmap package of RStudio software and considering the number of mutations found within this particular set of genes. The color and name of PDC variants for each of the isolates analyzed are shown. Download FIG S1, JPG file, 0.3 MB. Copyright © 2022 Colque et al. 2022 Colque et al. https://creativecommons.org/licenses/by/4.0/ This content is distributed under the terms of the Creative Commons Attribution 4.0 International license.

In order to understand the molecular bases for this phenotype, we performed a time-resolved tracking of mutations in *bla*_PDC_. With this aim, we obtained three collections, each including 30 isolates belonging to the same P. aeruginosa hypermutator lineage, from three successive sputum samples collected from patient CFD in 2011, 2012, and 2017. The *bla*_PDC_ gene was sequenced in all 90 isolates (Fig. 1A). As shown in Fig. 1A and C, the ancestor from 1991 and the 1995 isolate harbored the PDC-3 variant (37, 48). After 2 decades of chronic infection, 7 new *bla*_PDC_ allelic variants (referred to as PDC-458 to PDC-466) were identified in the 2011 collection. Each allele harbored 2 to 5 mutations relative to the ancestral *bla*_PDC-3_ gene, which were the result of different combinations of only 8 substitutions (Fig. 1D). Substitutions G214S and V329I in variant PDC-458 from the 2002 isolate were not present in the 2011 population, which instead displayed combinations of the other six substitutions distributed in six new PDC variants (Fig. 1D). PDC-462 (A89V, Q120K, V211A, N320S) was the most prevalent variant in the 2011 collection, being found in 14 (47%) coexisting isolates, followed by variant PDC-463 (A89V, Q120K, H188Y, V211A), which was present in 37% of the isolates. The four remaining allelic variants were rare and present in only one or two isolates (Fig. 1A and D).

Sequencing of the *bla*_PDC_ gene in 2012 and 2017 isolates revealed the coexistence of isolates harboring different PDC variants, which may have evolved under the treatment with ceftazidime during this period (Fig. 1A and B). The 2012 collection was dominated by PDC-462 (72%), and three new *bla*_PDC_ alleles (PDC-508, PDC-509 and PDC-458.1) were identified. PDC-508 seems to derive from PDC-462 through the addition of E185K, thus accumulating five substitutions. PDC-119 and PDC-509 represent single and double mutants, respectively, which share the G214S substitution present in PDC-458 (from the 2002 isolate), whereas PDC-509 contained the V211A substitution that was dominant among the PDC variants reported here. Although PDC-463 was present in high proportion in the 2011 collection, it was not detected a year later. PDC-461 (scarcely represented in the 2011 sampling) became prevalent in 2017 (39%), even overriding PDC-463 (36%), which was detected again in this collection. The occurrence of PDC-462 decreased from 71.8% in 2012 to 11% in the 2017 isolates. Two new *bla*_PDC_ alleles (PDC-465 and PDC-466) were detected in the 2017 sampling. PDC-465 showed the G214S substitution present in PDC-458, but combined with the novel T255P substitution. PDC-466, instead, seems to derive from PDC-462 through the addition of G204D, accumulating five substitutions. PDC-459, PDC-465, and PDC-466 were among the less prevalent variants in 2017 (Fig. 1A).

Overall, the main composition of *bla*_PDC_ mutations observed throughout 2011, 2012, and 2017 was conserved. A phylogenetic reconstruction of the evolutionary history of PDC reveals that two major clades emerged from the PDC-3 ancestor in 1991 (Fig. 1C). One clade is represented by the prevalent variants harboring the V211A substitution, and the second clade includes the less prevalent ones sharing the G214S substitution (Fig. 1C).

Next, we investigated whether these mutations were representative of the whole population of P. aeruginosa. Thus, to test the diversity and prevalence of *bla*_PDC_ mutations at the population level, we performed *bla*_PDC_ amplicon sequencing directly from DNA purified from the same sputum sample obtained from patient CFD in 2017 (Fig. S2). Following coverage analysis of >5,000 sequencing reads per base in the *bla*_PDC_ open reading frame, only mutations with population frequencies above 2% were considered (Table S2 at https://www.researchgate.net/publication/361224711_supplemental_material_for_1013140RG223345582088). The substitutions A89V, Q120K, V211A, and N320S were the most frequently observed changes, followed by H188Y and N346I (Fig. S2). The N346I substitution, which was not observed in any of the isolates previously analyzed, has been reported to confer resistance to expanded-spectrum cephalosporins (37). Substitutions G214S, T255P and G204D, present in PDC-465 or PDC-466, were not detected by this sequencing analysis, probably due to the low prevalence of these variants.

10.1128/mbio.01663-22.3 FIG S2 Population analysis of the CFD 2017 sample. The sputum sample collected from patient CFD was divided in two, and each half was processed for either Sanger sequencing of the *bla*_PDC_ gene in single isolates or sequencing of the *bla*_PDC_ gene directly amplified on the sputum sample, for which DNA was extracted and sequenced on Illumina MiSeq. The graphs represent the percentage of total reads of each amino acid (AA) variation in the whole population of P. aeruginosa (data not shown). T79A (gray bar), considered a polymorphism that does not affect β-lactam resistance (24), was found in 100% of the population, confirming the CFD patient was colonized by a P. aeruginosa lineage derived by a single ancestral clone containing the PDC-3 variant. Download FIG S2, TIF file, 0.1 MB. Copyright © 2022 Colque et al. 2022 Colque et al. https://creativecommons.org/licenses/by/4.0/ This content is distributed under the terms of the Creative Commons Attribution 4.0 International license.

This genetic analysis reveals that substitutions present in the most prevalent PDC variants in either 2011, 2012, or 2017 were the most frequent substitutions observed in the global population.

### Combinations of multiple *bla*_PDC_ mutations generate resistance to aztreonam and expanded-spectrum cephalosporins, including ceftolozane.

The chromosomally encoded PDC is overexpressed in clinical isolates, therefore contributing as the main mechanism of β-lactam resistance (15, 49). We performed MIC studies using ceftazidime and aztreonam in a series of CFD isolates expressing the mentioned PDC variants (Table S3 at https://www.researchgate.net/publication/361368769_Supplemental_Material_for_Longitudinal_evolution_of_the_Pseudomonas_Derived_Cephalosporinase_PDC_structure_and_3_activity_in_a_Cystic_Fibrosis_patient_treated_with_b-lactams). These determinations revealed a significant increase in the resistance against ceftazidime and aztreonam. We next tested the resistance of these isolates to ceftolozane-tazobactam. These latter experiments revealed an increase in the MICs from those of the 1991 ancestor, despite the fact this patient was never prescribed the combination of these drugs, which was approved in 2014 (Table S3 at https://www.researchgate.net/publication/361368769_Supplemental_Material_for_Longitudinal_evolution_of_the_Pseudomonas_Derived_Cephalosporinase_PDC_structure_and_3_activity_in_a_Cystic_Fibrosis_patient_treated_with_b-lactams). This poses the interesting question of whether ceftazidime administration may have provided a driving force for the development of resistance to ceftolozane. Since the evolved clinical isolates from the CFD patient have been reported to express high protein levels compared to the 1991 ancestor (47), and there are other factors that may lead to β-lactam resistance, we decided to explore the impact of these substitutions in PDC on the resistance of these P. aeruginosa isolates.

To dissect the role in β-lactam resistance of the mutations present in the different *bla*_PDC_ allelic variants, we designed a system to analyze resistance profiles in a common genetic background that allows the control of PDC expression levels. With this in mind, we selected P. aeruginosa PAO1 as the host for these experiments. This system enables us to address the specific impact of the mutations on the enzymatic activity, regardless of the expression levels.

First, a *bla*_PDC_-deficient PAO1 derivative strain (PAΔA) was constructed, in which the chromosomal *bla*_PDC_ gene was deleted. Then, selective *bla*_PDC_ allelic variants from the 26-year study representing the two major clades of the phylogeny (Fig. 1C) together with the ancestor PDC-3 variant from 1991 and the PDC-1 variant from the PAO1 strain were cloned into the pMBLe vector under the control of the *lac* operator (50) and transformed into PAΔA. As a control, we tested the antibiotic susceptibility and PDC levels of variants PDC-1 and PDC-458 at different concentrations of IPTG (isopropyl-β-d-thiogalactopyranoside) showing similar expression levels, as revealed by immunodetection at a concentration where MICs did not change (Fig. 1D; Fig. S3 and Table S4 at https://www.researchgate.net/publication/361368769_Supplemental_Material_for_Longitudinal_evolution_of_the_Pseudomonas_Derived_Cephalosporinase_PDC_structure_and_3_activity_in_a_Cystic_Fibrosis_patient_treated_with_b-lactams). Clones of PAΔA carrying different *bla*_PDC_ alleles were challenged against a panel of antipseudomonal β-lactam antibiotics commonly prescribed to CF patients (Table 1).

**TABLE 1:** Susceptibility profiles of the PAΔA strain complemented with the different PDC variants

Strain^*a*^	MIC (μg/mL)^*b*^								
	CAZ (R at ≥32 μg/mL)	CTZ (R at ≥16 μg/mL)	CTZ/TZ (R at ≥16/4 μg/mL)	AZT (R at ≥32 μg/mL)	PIP (R at ≥128 μg/mL)	PIP/TZ (R at ≥128/4 μg/mL)	FEP (R at ≥32 μg/mL)	IMP (R at ≥8 μg/mL)	MEM (R at ≥8 μg/mL)
PAO1	2	1	1	8	16	8	4	2	1
PAΔA	2	1	1	8	16	8	4	1	1
PAΔA-EV	1	1	1	4	4	4	2	0.5	0.5
PAΔA-1	4	1	1	8	64	16	4	1	1
PAΔA-3_(T79A)_	**4**	**1**	1	4	**64**	16	4	**1**	1
PAΔA-458_(G214S, V329I)_	4	2	1	16	16	8	2	1	1
PAΔA-459_(Q120K, P153L, V211A)_	128	32	8	128	32	16	4	1	1
PAΔA-460_(A89V, V211A, N320S)_	32	2	1	16	64	16	4	1	1
PAΔA-461_(A89V, Q120K, V211A)_	**128**	**16**	16	32	**8**	2	8	**1**	1
PAΔA-462_(A89V, Q120K, V211A, N320S)_	**128**	**8**	8	64	**64**	16	4	**1**	1
PAΔA-463_(A89V, H188Y, Q120K, V211A)_	**64**	**8**	4	128	**32**	16	4	**2**	1
PAΔA-464_(A89V, H188Y, Q120K, V211A, N320S)_	64	8	4	16	64	16	4	1	1

a *bla*_PDC_ allelic variants (PDC-3 and PDC-458 to PDC-464) were cloned into the pMBLe vector and transformed into the PAΔA strain. The PAΔA strain expressing the PDC variant from PAO1 (i.e., PAΔA-1) or the empty vector (i.e., PAΔA-EV) was used as a control.

b CAZ, ceftazidime; CTZ, ceftolozane; CTZ/TZ, ceftolozane-tazobactam at 2:1; AZT, aztreonam; PIP, piperacillin; PIP/TAZ, piperacillin-tazobactam at a fixed concentration of 4 μg/mL; FEP, cefepime; IMP, imipenem; MEM, meropenem. The resistance (R) thresholds are shown in parentheses for each drug or combination. Shown are values from at least two independent experiments. Values of MICs of PAΔA-3 and PAΔA-461 to -463 are highlighted in bold for comparisons with the kinetic parameters obtained for the CAZ, CTZ, PIP, and IMP antibiotics.

10.1128/mbio.01663-22.4 FIG S3 Representative Western blot showing the controlled expression of PDC variants PDC-1 and PDC-458. Entire cell extracts of strain PAΔA expressing either streptavidin-tagged PDC-1 or PDC-458 were used for Western blotting by using specific antibodies against streptavidin tag. Overnight bacterial cultures were induced with IPTG at 10 μM (+) or 20 μM (++) or grown without IPTG (−). Cell extracts of PAΔA transformed with the empty vector (EV) pMBLe or with no vector at all (ΔA) were used as negative controls. Download FIG S3, PDF file, 0.09 MB. Copyright © 2022 Colque et al. 2022 Colque et al. https://creativecommons.org/licenses/by/4.0/ This content is distributed under the terms of the Creative Commons Attribution 4.0 International license.

Bacteria expressing either PDC-3 or PDC-1 were susceptible to β-lactams, with the sole exception of piperacillin. The PDC-458 variant (from the 2002 isolate) resulted in higher levels of resistance to aztreonam. In contrast, the most representative allelic variants found in the 2011 and 2012 isolates (from PDC-459 to PDC-464) showed increased MICs against ceftazidime and aztreonam. These variants present different combinations of the A89V, Q120K, H188Y, P153L, V211A, and N320S substitutions, resulting in triple, quadruple, and quintuple substitutions. Some of these variants (PDC-459, PDC-461, and PDC-463) exhibited lower MICs against piperacillin and piperacillin-tazobactam, whereas none of them conferred resistance to cefepime or to the carbapenems imipenem and meropenem (Table 1).

All variants harboring the Q120K substitution (PDC-459 and PDC-461 to -464) showed high resistance levels to ceftolozane (susceptibility [S] at ≤4 μg/mL), either alone or combined with the β-lactamase inhibitor tazobactam (S at ≤4/2 μg/mL) (Table 1). These results support the notion that ceftolozane resistance is due (at least partially) to these substitutions in PDC. The highest resistance to both ceftazidime and ceftolozane was demonstrated by PDC-459 (including substitutions Q120K, P153L, and V211A), followed by PDC-461 (which clusters A89V, Q120K, and V211A). The addition of N320S and H188Y to the latter triple combination in PDC-462 and PDC-463, respectively, not only increased resistance to aztreonam, but also reverted the decrease in piperacillin resistance observed for PDC-461 (Table 1). On one hand, the lower resistance levels observed in a homogeneous genetic background reveal that the different expression levels in the isolates as well as other resistance mechanisms are working together in the clinical isolates. Importantly, this system enables us to directly assess the role of the observed mutations in the activity of the PDC variants.

### Differential competitiveness of coexisting PDC variants can shape the dynamics of resistant subpopulation of P. aeruginosa upon exposure to β-lactams.

The effect of multiple combined *bla*_PDC_ mutations on bacterial fitness was explored by performing competitive growth assays using the P. aeruginosa PAΔA strain carrying the most prevalent *bla*_PDC_ allelic variants among the CFD 2011, 2012, and 2017 populations (referred to as PAΔA-461, PAΔA-462, and PAΔA-463), tagged with the *lacZ* gene. We also analyzed PAΔA-464, despite its low abundance. This allelic variant combined A89V, Q120K, H188Y, V211A, and N320S substitutions (Fig. 1D). Growth curves did not reveal any deleterious effect due to gene expression in any of these constructs (Fig. S4). Pairs of tagged/untagged strains were cocultured *in vitro* and then plated on LB agar plates containing X-Gal (5-bromo-4-chloro-3-indolyl-β-d-galactopyranoside).

10.1128/mbio.01663-22.5 FIG S4 Growth curves of PAΔA expressing each of the PDC variants. Strains were grown in LB broth supplemented with 10 μM IPTG at 37°C for 16 h. Experiments were performed in duplicate, and mean values are shown. No significant differences were observed; therefore, error bars are omitted for clarity. Download FIG S4, PDF file, 0.1 MB. Copyright © 2022 Colque et al. 2022 Colque et al. https://creativecommons.org/licenses/by/4.0/ This content is distributed under the terms of the Creative Commons Attribution 4.0 International license.

**FIG 2:**
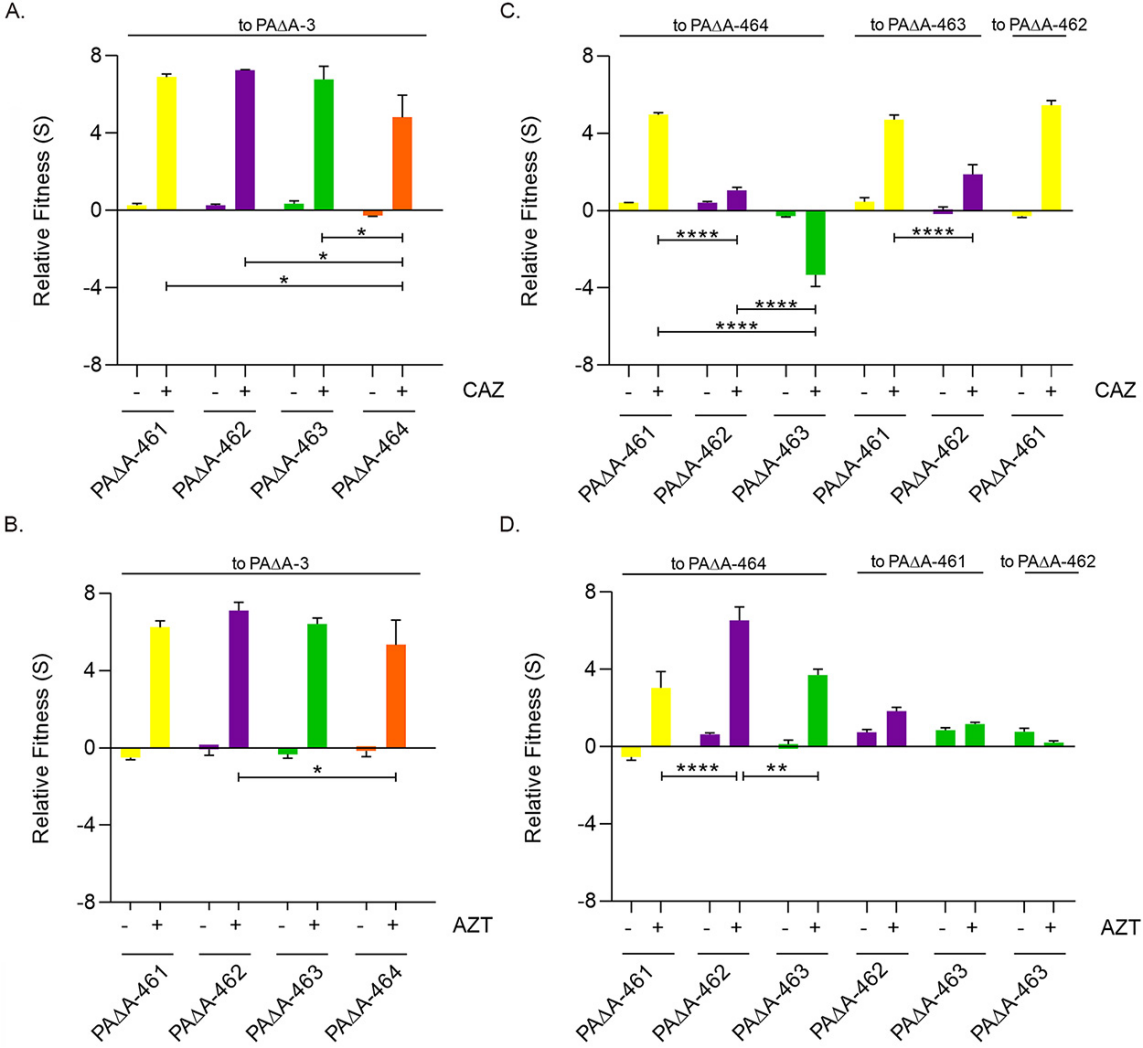
*In vitro* competition experiments among different *bla*_PDC_ alleles. Competition experiments were performed in the absence or presence of ceftazidime (CAZ [A and C]) or aztreonam (AZT [B and D]). PAΔA strains expressing PDC-461, PDC-462, PDC-463, or PDC-464 (i.e., PAΔA-461, PAΔA-462, PAΔA-463, and PAΔA-464, respectively) were competed against PAΔA expressing PDC-3 (PAΔA-3). Fitness (S) relative to PAΔA-3 for (A) ceftazidime or (B) aztreonam is shown. Then, PAΔA-461, PAΔA-462, PAΔA-463, and PAΔA-464 were competed against each other in the absence of antibiotics or presence of (C) ceftazidime or (D) aztreonam. (See Text S1 in the supplemental material for the antibiotic concentration scheme.) Measurements were carried out in triplicate for at least two independent experiments, and the results are expressed as means ± standard errors of the means (SEM). Statistically significant differences at *P* < 0.0001, *P* < 0.01, and *P* < 0.05 are identified by ****, **, and *, respectively (two-way analysis of variance [ANOVA] followed by Tukey’s multiple-comparison test).

We first evaluated the relative fitness by analyzing the competition of each variant with the PAΔA strain expressing the ancestral PDC-3 (PAΔA-3). As shown in Fig. 2A and B, significant differences were not observed in the absence of antibiotics. Instead, in the presence of ceftazidime or aztreonam (the antibiotics used in the therapy of patient CFD), PAΔA-461, PAΔA-462, PAΔA-463, and PAΔA-464 clearly outcompeted PAΔA-3. PAΔA-464 showed lower levels of competitiveness than PAΔA-461, PAΔA-462, and PAΔA-463, indicating that the introduction of a fifth substitution compromises resistance to these antibiotics.

10.1128/mbio.01663-22.1 TEXT S1 Supplemental materials and methods. Download Text S1, DOCX file, 0.09 MB. Copyright © 2022 Colque et al. 2022 Colque et al. https://creativecommons.org/licenses/by/4.0/ This content is distributed under the terms of the Creative Commons Attribution 4.0 International license.

We also performed a pairwise competition of the evolved variants in the presence of ceftazidime. PAΔA-461 showed a clear advantage compared to PAΔA-462, PAΔA-463, and PAΔA-464 (Fig. 2C), suggesting that the A89V-Q120K-V211A combination provides the highest relative fitness, whereas additional substitutions impaired competitiveness in the presence of this antibiotic. To illustrate, the quadruple variant PAΔA-462 showed higher fitness than PAΔA-463 (harboring H188Y instead of N320S) and PAΔA-464 (harboring both H188Y and N320S), which in turn outcompeted PAΔA-463 (Fig. 2C).

In the presence of aztreonam, a clear fitness advantage was observed for all variants against PAΔA-464. PAΔA-463 and PAΔA-462 showed higher relative fitness than PAΔA-461, suggesting that the addition of N320S and H188Y extends the spectrum of β-lactam resistance (Fig. 2D).

### PDC variants show improved hydrolytic activity toward ceftazidime and ceftolozane.

We next assessed the capacity of the most relevant PDC variants to hydrolyze β-lactams. The mature PDC-3, PDC-461, PDC-462, and PDC-463 proteins were expressed and purified from E. coli cultures to homogeneity. Then, we performed steady-state kinetic measurements to test the catalytic efficiencies against the β-lactams ceftazidime, ceftolozane, piperacillin, and imipenem. In agreement with previous reports (45, 48, 51), PDC-3 hydrolyzed efficiently piperacillin while showing a poor hydrolytic activity against ceftazidime, ceftolozane, and imipenem (Table 2).

**TABLE 2:** Kinetic parameters of PDC variants with different β-lactam substrates^*a*^

β-Lactam	PDC-3			PDC-461			PDC-462			PDC-463		
	*K_m_* (μM)	*k*_cat_ (s^−1^)	*k*_cat_/*K_m_* (mM^−1^ s^−1^)	*K_m_* (μM)	*k*_cat_ (s^−1^)	*k*_cat_/*K_m_* (mM^−1^ s^−1^)	*K_m_* (μM)	*k*_cat_ (s^−1^)	*k*_cat_/*K_m_* (mM^−1^ s^−1^)	*K_m_* (μM)	*k*_cat_ (s^−1^)	*k*_cat_/*K_m_* (mM^−1^ s^−1^)
CAZ	57.4 ± 39.3	0.0105 ± 0.000778	0.183 ± 0.138	93.1 ± 40.5	0.465 ± 0.00707	4.99 ± 2.25	23.1 ± 1.98	0.122 ± 0.0103	5.28 ± 0.897	55.0 ± 26.5	0.133 ± 0.0106	2.41 ± 1.35
PIP	18.6 ± 6.84	2.86 ± 1.93	154 ± 160	105 ± 1.55	0.145 ± 0.00705	1.38 ± 0.0871	9.86 ± 3.23	1.44 ± 0.0574	146 ± 5.38	54.5 ± 34.8	0.801 ± 0.216	14.7 ± 1.33
IMP	10.4 ± 2.96	0.0159 ± 0.00872	1.53 ± 1.27	33.9 ± 21.4	0.00725 ± 0.00247	0.214 ± 0.208	37.3 ± 3.62	0.0389 ± 0.00512	1.03 ± 0.238	35.9 ± 4.76	0.0130 ± 0.00141	0.362 ± 0.0872
CTZ^*b*^	ND	ND	0.040 ± 0.0092	ND	ND	6.1 ± 1.3	ND	ND	0.66 ± 0.14	ND	ND	0.33 ± 0.055

a Kinetic parameters were determined for ceftazidime (CAZ), piperacillin (PIP), imipenem (IMP), and ceftolozane (CTZ). Two independent experiments were carried out, and results are expressed as means ± SEM.

b Due to the low hydrolysis of ceftolozane, *k*_cat_/*K_m_* values were obtained from Lineweaver-Burk plots. ND, not determined.

PDC-461, -462, and -463 efficiently hydrolyzed ceftazidime, showing 28-, 29-, and 13-fold increased catalytic efficiencies, respectively, relative to the ancestor PDC-3, indicating that mutations in these PDCs improved their catalytic performance against this cephalosporin. PDC-461 displayed a catalytic efficiency against piperacillin 100-fold impaired with respect to PDC-3, disclosing a trade-off in the substrate profile shaped by the presence of the three core mutations (A89V, Q120K, and V211A). Instead, the additional mutations present in PDC-462 and PDC-463 were able to restore this activity. Indeed, PDC-462 displayed hydrolytic levels against piperacillin similar to those of PDC-3, in agreement with the observed piperacillin MICs for the strain expressing this variant (Table 1).

All PDC variants maintained very low hydrolysis rates for imipenem, displaying *k*_cat_ values between 0.01 and 0.04 s^−1^, which correlate well with the imipenem susceptibility (MICs of 1 to 2 μg/mL) observed in PAΔA expressing either PDC-461, -462, or -463 variants (Table 1). Remarkably, when the catalytic efficiencies of PDC-461, -462, and -463 were assessed against the recently introduced cephalosporin ceftolozane, the *k*_cat_/*K*_m_ ratios showed significantly increased values compared to that of the parental enzyme. PDC-462 and -463 show 16- and 8-fold enhancements of this activity, a performance that is largely overcome by PDC-461, showing a 150-fold increase in *k*_cat_/*K*_m_.

These catalytic efficiencies correlate very well with the MIC determinations of different antibiotics determined in an isogenic Pseudomonas background (Table 1), revealing that the different accumulated mutations are responsible for tuning the substrate profile of these variants, including the newest cephalosporin, ceftolozane.

### Molecular modeling simulations reveal enlargement of the substrate binding pocket in PDC evolved mutants.

**FIG 3:**
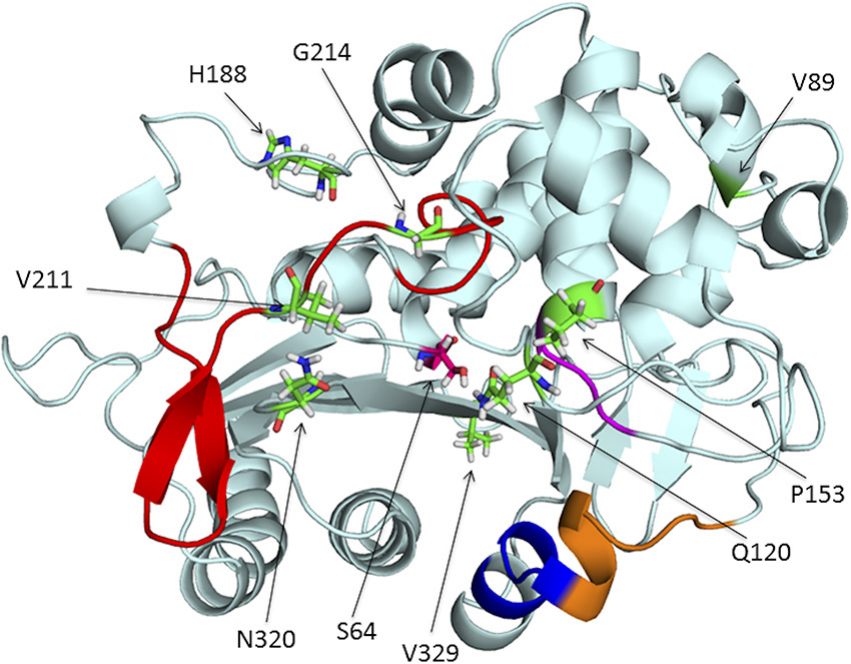
Representation of the PDC β-lactamase structure from P. aeruginosa PAO1 (PDB 4OOY). The different structural regions lining the binding site are colored as follows: Ω-loop, red; helix H-10, blue; R2 loop, orange; YSN, purple. The amino acid residues that were mutated across the different *bla*_PDC_ allelic variants in this study are represented with sticks in green and pointed at with arrows.

To understand the role of the different mutations (Fig. 3) in the catalytic activity of PDC-3, we resorted to computational chemistry studies. The different substitutions present in the studied variants are scattered in the protein structure, many of them being part of protein loops (Fig. 3). Expansion of the substrate profile by mutations in β-lactamases has been accounted for by changes in the protein dynamics. Therefore, we first performed 200-ns classical molecular dynamics (MD) simulations on these variants (PDC-3, PDC-461, PDC-462, and PDC-463) in the unbound state.

All proteins preserved their global tertiary structure during the MD simulations as stems from inspecting the evolution of the root mean square deviation (RMSD) over time (Fig. S5A). After an equilibrium time, the values stabilize in similar values for all the enzyme variants. In the same way, the root mean square fluctuation (RMSF) study shows us that the fluctuations related to the different regions of the enzymes are conserved in all variants (Fig. S5B).

10.1128/mbio.01663-22.6 FIG S5 RMSD (A) and RMSF (B) values calculated during the MD simulations of the different proteins studied: PDC-3 (gray), PDC-461 (yellow), PDC-462 (purple), PDC-463 (green). Download FIG S5, PDF file, 0.1 MB. Copyright © 2022 Colque et al. 2022 Colque et al. https://creativecommons.org/licenses/by/4.0/ This content is distributed under the terms of the Creative Commons Attribution 4.0 International license.

**FIG 4:**
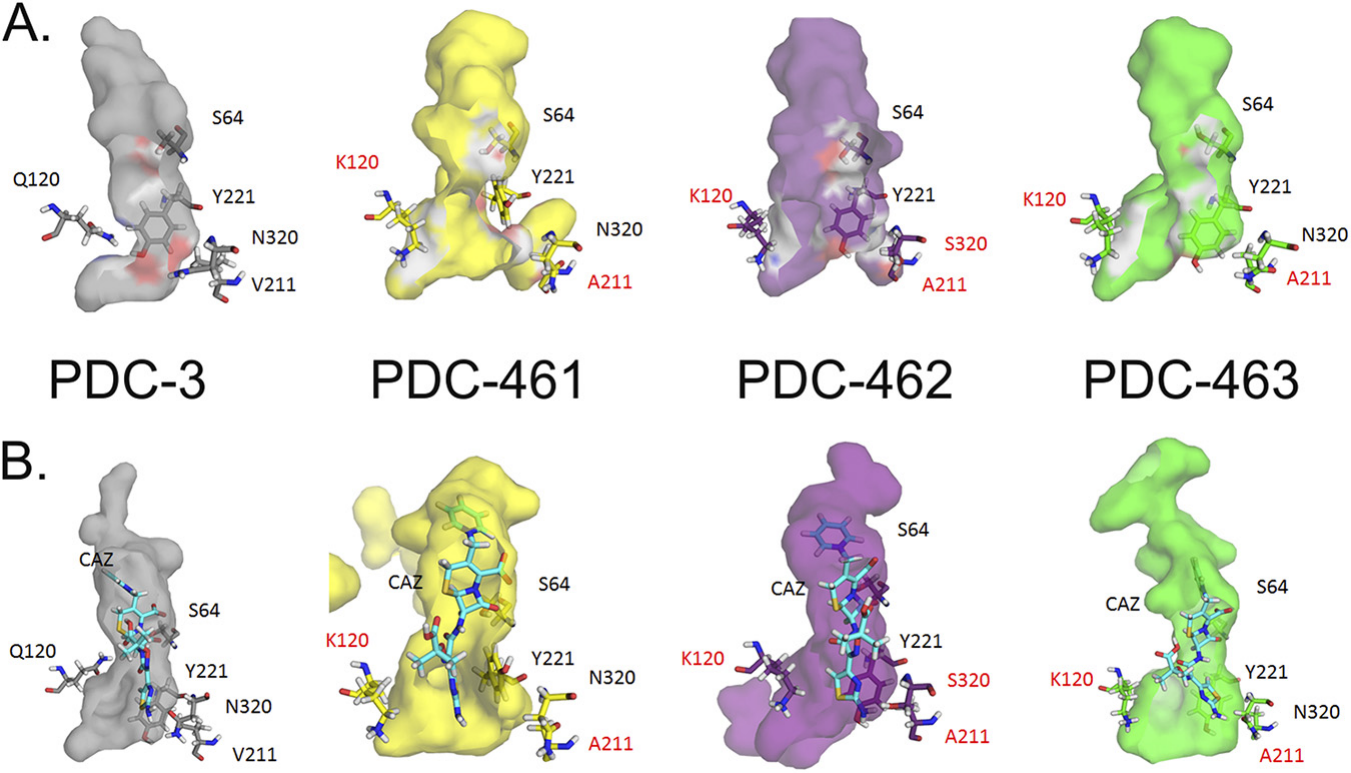
Molecular modeling of PDC proteins. The structures of the apo/free versions (A) and those coupled with ceftazidime antibiotic CAZ (B) with their active site cavities are shown. Colors of protein structures are as follows: PDC-3, gray; PDC-461, yellow; PDC-462, purple; PDC-463, green.

**FIG 5:**
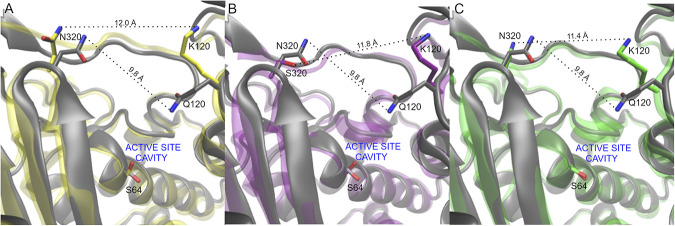
Comparison of representative snapshots of the MD simulations of each protein studied compared to PDC-3 showing the active site cavity environment (R1 region). In all representations, N atoms are depicted in blue, O atoms are depicted in red, and key residues are highlighted in sticks. C atoms of the PDC-3 are depicted in gray. (A) PDC-3 versus PDC-461 (C atoms in yellow); (B) PDC-3 versus PDC-462 (C atoms in purple); (C) PDC-3 versus PDC-463 (C atoms in green). Residue distances are depicted with dashed lines.

A more in-depth analysis of protein dynamics reveals significant changes in the substrate binding pocket elicited by the substitutions (Fig. 4). The three variants showing an enhanced activity toward ceftazidime and ceftolozane (PDC-461, PDC-462, and PDC-463) present a broader active site cavity. Figure 5 shows how the different mutations enlarge the cavity of the active site in the R1 region. The distance in a representative snapshot of the MD simulation from N320 to Q120 in PDC-3 is approximately 9.8 Å, while in the different variants, the distances between N320 and K120 are 12.0 Å in PDC-461 and 11.4 Å in PDC-463. In the PDC-462 variant, the distance between S320 and K120 is around 11.8 Å. For a better illustration of this enlargement, we plot the distances mentioned as a function of time of the MD simulation in Fig. S6.

10.1128/mbio.01663-22.7 FIG S6 Distances as a function of time during 200-ns MD simulations are shown for PDC-3 (A), PDC-461 (B), PDC-462 (C), and PDC-463 (D). The distance is measured as indicated in the figure prot-dist-prom (Fig 5) between Nδ of N320, Oγ of S320, Nε of Q120, and Nζ of K120 according to the variant studied. The average distance in each case is plotted with solid black line. Download FIG S6, TIFF file, 0.3 MB. Copyright © 2022 Colque et al. 2022 Colque et al. https://creativecommons.org/licenses/by/4.0/ This content is distributed under the terms of the Creative Commons Attribution 4.0 International license.

**FIG 6:**
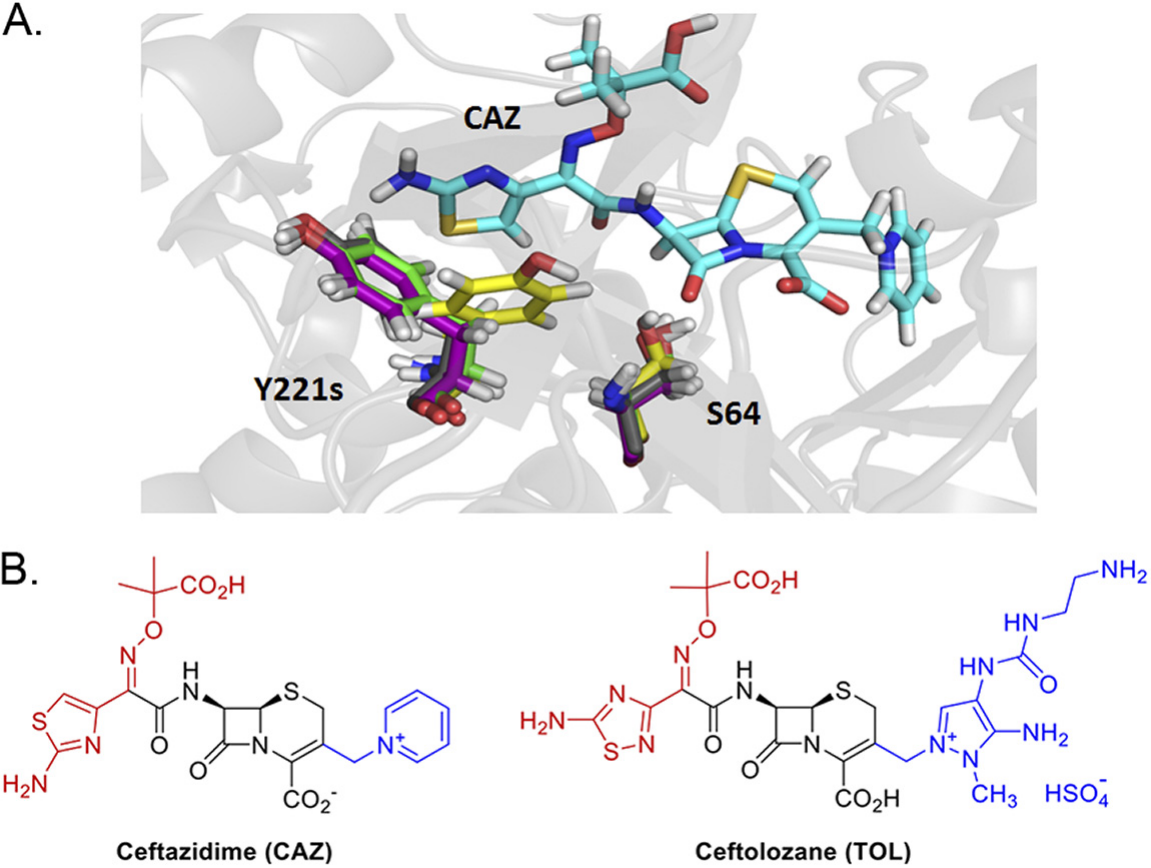
Orientation change in the Y221 residue and evidence of a stacking interaction. (A) When ceftazidime (CAZ) antibiotic was introduced in PDC protein structure modeling, a strong orientation change in the Y221 residue of PDC-461 was seen. Y221 residues are colored in gray, yellow, purple, and green as for their PDC variants PDC-3, PDC-461, PDC-462, and PDC-463, respectively. The structure of the CAZ antibiotic and the location of the S64 active site are also depicted. (B) Structures of β-lactam antibiotics for which PDC variants showed increased resistance. The R1 side chains are shown in red, and the R2 side chains are shown in blue.

By visual inspection of the MD simulations, we observed that the substitution V211A induces a conformational change in the Ω-loop, involving residues 200 to 223. As a result, a hydrogen bond formed by phenolic OH of Y221 with the backbone of G214 present in PDC-3 is lost, inducing a conformational change in Y221 in PDC-461 (Fig. 6). Mutation Q120K eliminates a hydrogen bonding interaction of the amide side chain with N152 (from the YSN loop, located on the opposite side of the substrate binding pocket). This results in a conformational change of this residue, with K120 pointing outwards and therefore further widening the active site cavity (Fig. 5). The structural impacts of this mutation are similar in PDC-461, -462, and -463 (i.e., regardless of the genetic background), highlighting the key role of the Q120K mutation in the evolution of resistance.

To complement the study of the different variants in the unbound state, we resorted to quantum mechanics-molecular mechanics (QM-MM) to gain further insight on the interactions of the different variants with ceftazidime. Representative conformations extracted from the MD simulations were used to build *in silico* the complexes of each PDC variant with ceftazidime, using the crystal structure of acylated ceftazidime bound to EDC (Escherichia coli*-*derived cephalosporinase) (PDB 1IEL) (52) as a template. In all four complexes, ceftazidime interacts with residues S64, K67, N152, Y221, S318, N320, N343, and N346, in agreement with the previous structural information (45, 52). The active site changes in the mutants allow a better accommodation of the bulky R1 side chain from ceftazidime (Fig. 4B; Fig. S7). In addition, the conformational change of Y221 results in an aromatic stacking interaction with the 4-thiazolyl ring of ceftazidime at the R1 substituent (Fig. 6). The N320S substitution in PDC-462 removes a hydrogen bond with A211 at the Ω-loop, which enables it to recover the interaction between Y221 and G214. Overall, all variants show a broadening of the active site that accounts for the large increase in activity and resistance against ceftazidime of these PDC variants.

10.1128/mbio.01663-22.8 FIG S7 Representation of the volume of the active site cavity calculated in a representative snapshot of the MD simulations overlaid with the complex protein-ceftazidime optimized structures for each protein studied. N atoms are depicted in blue, O atoms in red, C atoms of the protein in cyan, and C atoms of ceftazidime in orange. The volume of the cavity is represented with a surface shape representation in different colors: (A) PDC-3, gray; (B) PDC-461, yellow; (C) PDC-462, purple; (D) PDC-463, green. Download FIG S7, TIFF file, 3.4 MB. Copyright © 2022 Colque et al. 2022 Colque et al. https://creativecommons.org/licenses/by/4.0/ This content is distributed under the terms of the Creative Commons Attribution 4.0 International license.

## DISCUSSION

From our previous studies of the evolution of P. aeruginosa hypermutator lineages combining longitudinal and cross-sectional analysis covering decades of CF chronic infection, we showed that antibiotic resistance increases as infection progresses toward the establishment of a highly diversified population, that converges toward multidrug resistance (27, 47). Genes involved in β-lactam resistance were simultaneously mutated, indicating they are involved in the higher resistance levels of the CF isolates (47). In particular, two genes were among the most repeatedly altered by mutations: the gene coding for the chromosomal β-lactamase PDC and the gene coding for the penicillin binding protein PBP3, both linked to cephalosporin and carbapenem resistance, respectively. In this work, we dissect the specific impact of the mutations accumulated in the *ampC* gene (*bla*_PDC_) on the evolution of β-lactam resistance.

To our knowledge, few investigations have examined the evolution of PDC across P. aeruginosa populations over the course of long-term treatment *in vivo*. To address this gap and to better understand what is occurring in individual patients, we focused on a single patient, expanding the analyses over the course of 26-years of a P. aeruginosa hypermutator lineage in the CF lung. Despite the fact that single sputum samples might not be fully representative of the whole complexity of the different populations inhabiting different lung regions in the CF airways, this study tracks the same P. aeruginosa lineage, analyzing the resistance phenotypic diversification along 26 years of chronic infection.

Here, we show how the adaptive evolution of the ancestor PDC-3 variant in response to the prolonged and continuous antibiotic treatment evolved through the accumulation and selection of multiple mutations *in bla*_PDC_. In particular, we report a large increase in the hydrolytic capability of variants PDC-461, PDC-462, and PDC-463 against ceftazidime and ceftolozane, providing a structural and functional rationale. Despite these substitutions that improve the catalytic efficiency of PDC, it is clear that there are other mechanisms contributing to β-lactam resistance, such as the overexpression of these enzymes and mutations of other genes in the β-lactam resistome.

The high efficiency acquired by these PDC variants to confer resistance to the novel antipseudomonal cephalosporin ceftolozane is of great concern. Resistance to ceftolozane has previously been observed in P. aeruginosa*-*infected patients treated with this antibiotic (40, 53), and other studies have demonstrated that expression of a PDC-3 variant carrying a single E219K mutation can confer high MICs of ceftolozane in E. coli (45). The substitution of D217 at the Ω-loop, selected after treating a multidrug-resistant P. aeruginosa strain with ceftolozane-tazobactam, also results in an enhanced resistance toward this cephalosporin–β-lactamase inhibitor combination (46). *In vitro* long-term experiments with wild-type and mutator strains of P. aeruginosa exposed to increasing concentrations of ceftolozane-tazobactam showed that only mutator strains were able to develop high levels of resistance, by acquiring multiple mutations that led to overexpression and structural modifications of PDC (35). Here, we provide an example of the development of collateral resistance to ceftolozane in a patient who was never treated with this antibiotic. The overall impact of an increased mutational frequency caused by hypermutability is clearly manifested in this analysis (17, 54, 55).

In addition to diverse polymorphisms previously described for PDC, we report three new amino acid substitutions—A89Y, G204D, and T255P—which together with Q120K, P153L, E185K, H188Y, G214S, N320S, V329I, and N346I generated novel *bla*_PDC_ allelic variants, each harboring from 2 to 5 mutations. P153L, V211A, and N346I are located next to the conserved YSN loop, the C-terminal region of the Ω-loop, and the C3/C4 recognition region, respectively, and have been shown to individually confer resistance to β-lactam antibiotics in P. aeruginosa clinical isolates (37, 45). The finding of new combinations that further enhance cephalosporin resistance supports the adaptability of the PDC scaffold to tolerate various mutations, which at the same time provides a substantial gain of function. These observations will also serve as a starting point for future mechanistic studies.

The three most prevalent alleles include mutations A89V, Q120K, and V211A and, at the same time, confer the highest resistance to cephalosporins and enhanced competitiveness in the presence of ceftazidime. Molecular dynamics simulations revealed that these three mutations give rise to a wider substrate binding pocket in the active site. This structural change provides space for better accommodating the R1 side chain of ceftazidime, improving binding of this antibiotic to the enzyme (Fig. 4 and Fig. 6A). In addition, substitutions Q120K and V211A trigger a different spatial orientation of the aromatic group of Y221 in the PDC-461 variant, favoring a stacking interaction between Y221 and the 4-thiazolyl ring present in the R1 group of ceftazidime (Fig. 6). Other substitutions located in or near the Ω-loop have been shown to enhance cephalosporin resistance by altering the conformation of Y221 (45, 52, 56). In the PDC variants herein described, this conformational change improves hydrolysis of ceftazidime and ceftolozane, but has the opposite effect on piperacillin. Instead, the N320S substitution in variant PDC-462 restores the Y221 orientation present in PDC-3, while maintaining the enlargement of the substrate binding pocket, thus extending the hydrolysis toward ceftazidime, ceftolozane, aztreonam, and piperacillin. These results suggest that the broadening of the active site induced by the different mutations is more relevant than the interaction of substrates with Y221.

The Q120K mutation results in a net widening of the active site, due to a conformational change of its side chain (Fig. 5). The impact of Q120K on resistance is evident from analyzing PDC-462 and PDC-460, which differ only by this substitution. The presence of this substitution elicits a 3-fold increase in the MICs of ceftazidime, aztreonam, and ceftolozane (Table 1). We conclude that Q120K plays an important role in the evolution of resistance in these variants.

All of these structural changes result in a better accommodation of the R1 group from ceftazidime: the volume in the active site region that recognizes R1 is increased by more than 2-fold in PDC-461, PDC-462, and PDC-463 compared to that in PDC-3 (Fig. 4). Interestingly, the R1 group structures, which have been associated with the antipseudomonal activity of cephalosporins (57), are almost identical in ceftazidime and ceftolozane (Fig. 6B). In contrast, changes in the volume of the active site cleft where R2 is positioned are not determinants for the binding of ceftazidime. We conclude that optimization of the binding pocket to accommodate the R1 side chain in ceftazidime elicits a better recognition of ceftolozane. Berrazeg and coworkers (37) have proposed that either ceftazidime or cefepime could induce this effect. In light of the current study, we conclude that the smaller R1 group in cefepime (compared to ceftazidime and ceftolozane) may elicit a similar cross-resistance effect. These observations and analyses explain the impact on *K_m_*; more advanced studies beyond the scope of this analysis will be needed to understand catalytic turnover.

In conclusion, this study combined genetic, biochemical, and structural analyses to assess the evolutionary processes driving how PDC β-lactamase confers resistance, which occurred throughout more than two and a half decades of CF chronic airway infections. This detailed scrutiny of the evolution of a P. aeruginosa clone persisting within a single patient reveals how consistent and intensive antibiotic treatment in the setting of a hypermutator genotype leads to a multidrug resistance phenotype, primarily driven by combined substitutions in the *bla*_PDC_ gene. The amazing plasticity of the PDC structure not only confirms the already known capacity to evolve when facing the challenge of new β-lactams, but also warns us that (possibly) chemical similarities among β-lactams from different generations could lead to an unexpected evolution of resistance, particularly in the context of a chronic infection by a hypermutator strain. Altogether, our results emphasize the huge evolutionary potential and impact of hypermutator strains and uncover the link between the antibiotic prescription history and the within-patient evolution of antibiotic resistance that relies on a molecular-based hypothesis of the adaptation of the PDC β-lactamase. Finally, our results highlight the importance of integrating bench-to-bedside research to fully understand the processes that lead to antibiotic resistance.

## MATERIALS AND METHODS

Clinical isolates were obtained from sputum samples from an adult patient with cystic fibrosis attending the Copenhagen Cystic Fibrosis Center at University Hospital Rigshospitalet, Denmark (CFD patient) (27). The use of the samples was approved by the local ethics committee of the Capital Region of Denmark (Region Hovedstaden; registration no. H-A-141 and H-1-2013-032), and the patient gave informed consent. Sputum samples were processed a median of 2 days after expectoration. During the lag time between expectoration and processing, the samples were stored at 4°C.

Isolation and identification of P. aeruginosa from sputum were carried out as previously described (58). The patient’s age at the time of the first isolate collection was 23 years, and the onset of the chronic infection with P. aeruginosa was in 1986. The P. aeruginosa collection included an initial isolate from 1991, two intermediate isolates from 1995 and 2002, and three populations of isolates collected in 2011 (27), 2012 and 2017 (this study). For cross-sectional analysis, sputum samples were liquefied by addition of an equal volume of Sputolysin (Calbiochem), homogenized, serially diluted, and plated onto Pseudomonas isolation agar (BD Biosciences). Then, 30 isolates were taken randomly to obtain the 2011, 2012, and 2017 collections. Isolates were stored at −70°C in glycerol stock solution. The 2017 sputum sample was divided in two: one part for the isolation of P. aeruginosa clones and the other for DNA extraction for ultradeep sequencing analysis.

A description of the materials and methods for sequence analysis of the *bla*_PDC_ gene in P. aeruginosa CFD isolates, DNA extraction and PCR amplification of the *bla*_PDC_ gene from whole sputum samples, the construction of P. aeruginosa Δ*bla*_PDC_-deficient (PAΔA) and *bla*_PDC_-*lacZ* (PAΔA-*lacZ*) strains, cloning of *bla*_PDC_ allelic variants and determination of expression levels of PDCs in the pMBLe vector, competition experiments for determination of competitive fitness of *bla*_PDC_ variants, expression and purification of PDC proteins, classical molecular dynamic simulations, and QM-MM calculations with the PDC variants (in apo versions) and PDC in complex with ceftazidime are provided in detail in Text S1 in the supplemental material.

### Data availability.

The sequences of the *bla*_PDC_ gene corresponding to the described PDC variants have been deposited in GenBank at https://www.ncbi.nlm.nih.gov/genbank/ under the accession numbers shown in parentheses: BankIt2402117 PDC-458 (MW287261), BankIt24021347 PDC-459 (MW287262), BankIt2402138 PDC-460 (MW287263), BankIt2402139 PDC-461 (MW287264), BankIt2402140 PDC-462 (MW287265), BankIt2402141 PDC-463 (MW287266), BankIt2402142 PDC-464 (MW287267), BankIt2402144 PDC-465 (MW287268), BankIt2402146 PDC-466 (MW287269), PDC-508 (OL989851), PDC-509 (OL989853), and PDC-119 (OL989852). Ultradeep sequencing data are available in the BioProject database at https://www.ncbi.nlm.nih.gov/bioproject/?term=PRJNA729779 under accession no. PRJNA729779.
