# Supplementary figures and images for: Longitudinal Evolution of the Pseudomonas-Derived Cephalosporinase (PDC) Structure and Activity in a Cystic Fibrosis Patient Treated with β-Lactams
Source: mBio. 2022 Sep 8;13(5):e01663-22. doi: 10.1128/mbio.01663-22 (PMC9600753; doi:10.1128/mbio.01663-22)

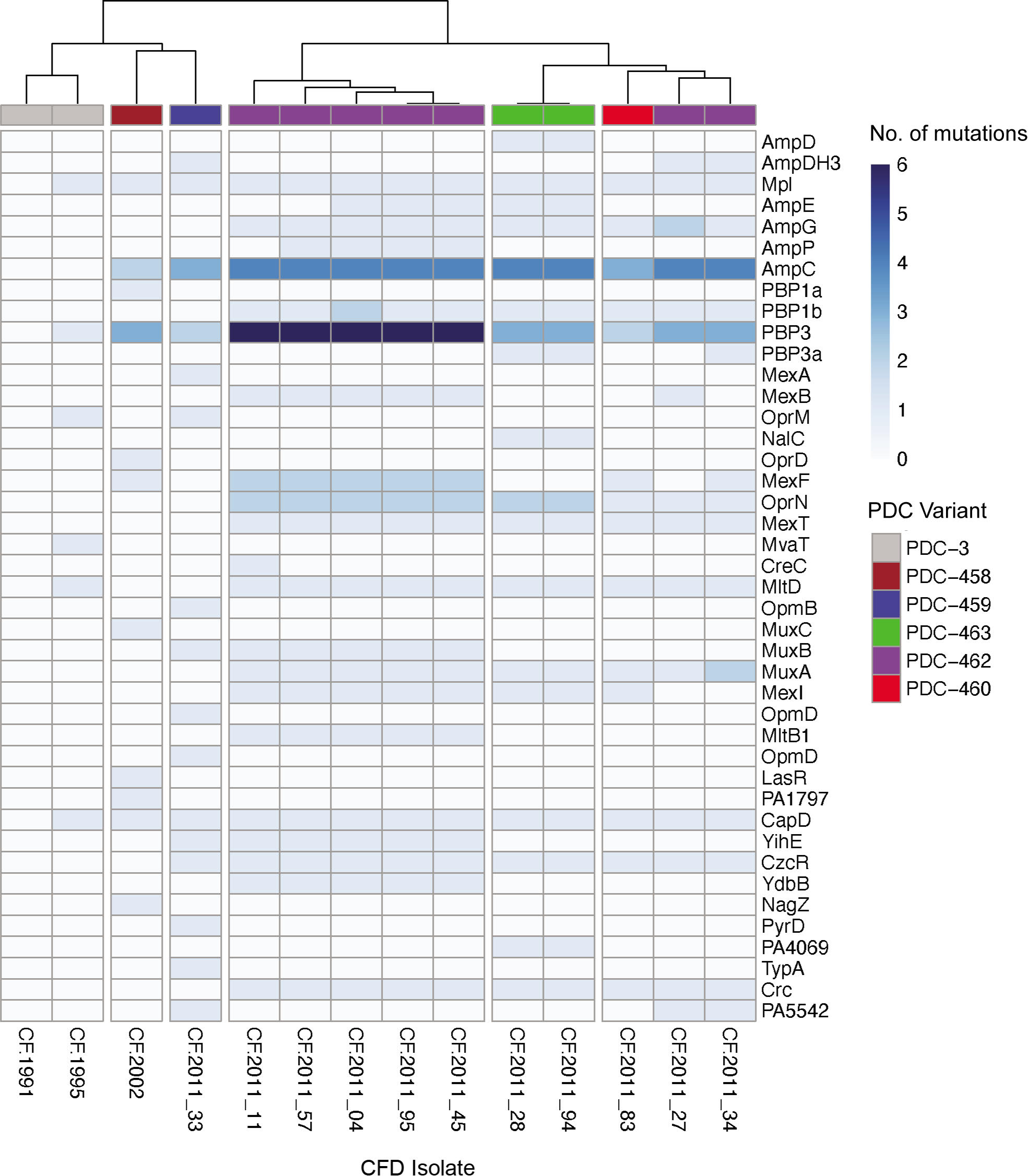

Supplement: FIG S1 [file mbio.01663-22-s0001.jpg]

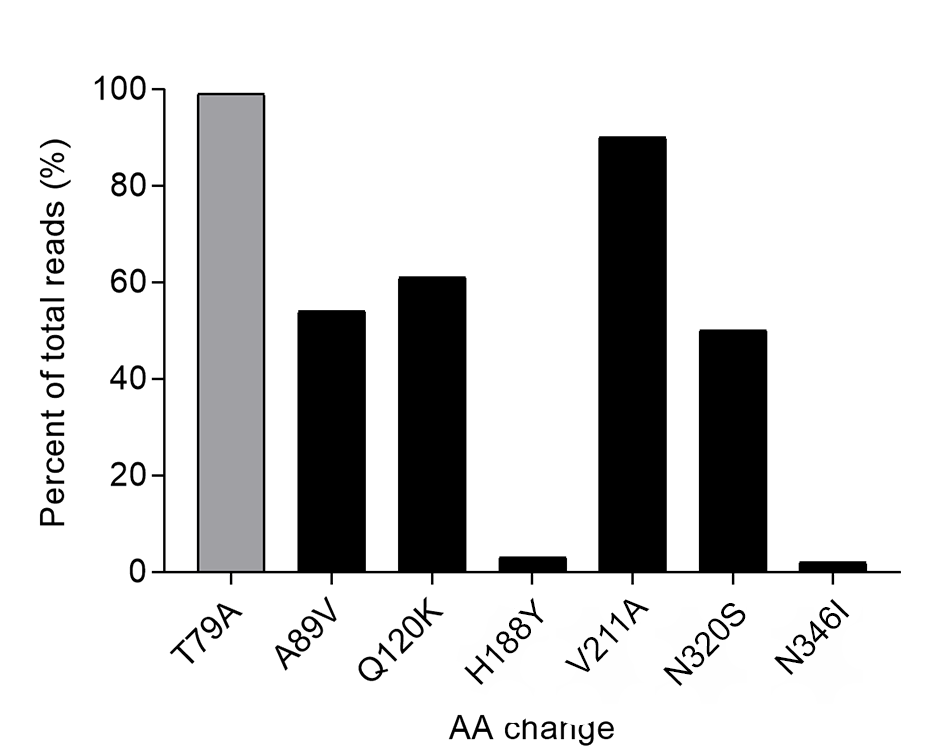

Supplement: FIG S2 [file mbio.01663-22-s0002.tif]

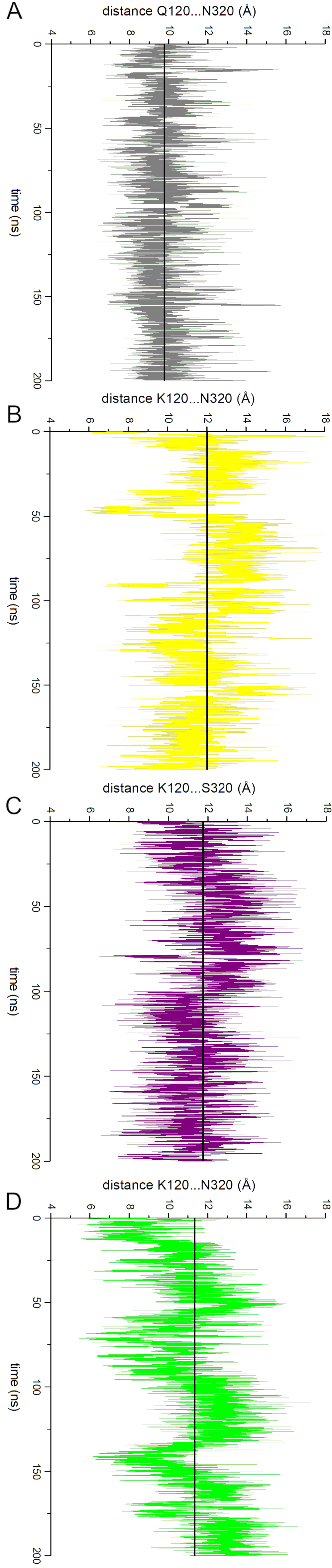

Supplement: FIG S6 [file mbio.01663-22-s0006.tiff]

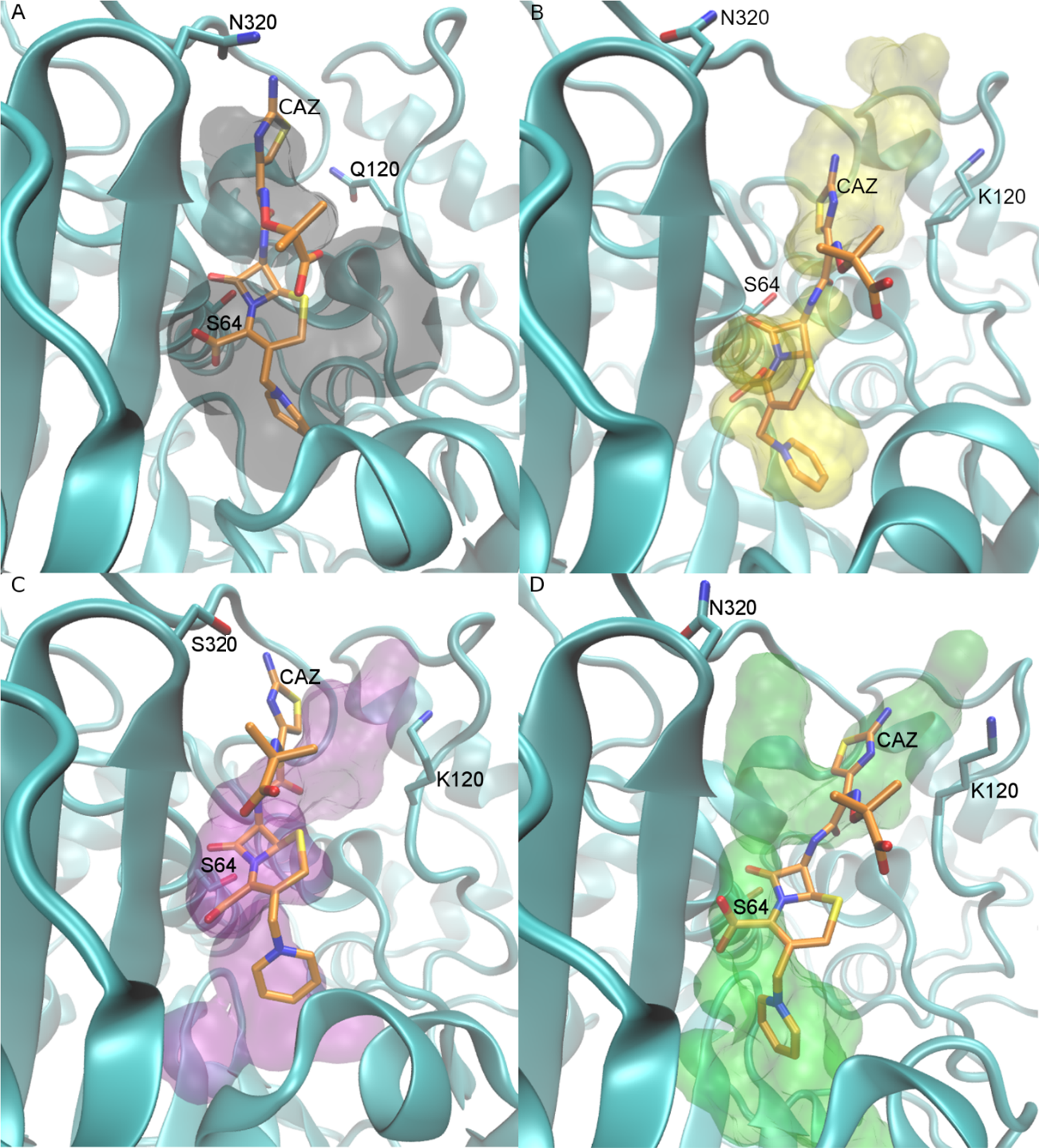

Supplement: FIG S7 [file mbio.01663-22-s0007.tiff]
