# Supplementary material for: Longitudinal Evolution of the Pseudomonas-Derived Cephalosporinase (PDC) Structure and Activity in a Cystic Fibrosis Patient Treated with β-Lactams
Source: mBio. 2022 Sep 8;13(5):e01663-22. doi: 10.1128/mbio.01663-22 (PMC9600753; doi:10.1128/mbio.01663-22)
Supplement: FIG S3 [file mbio.01663-22-s0003.pdf]

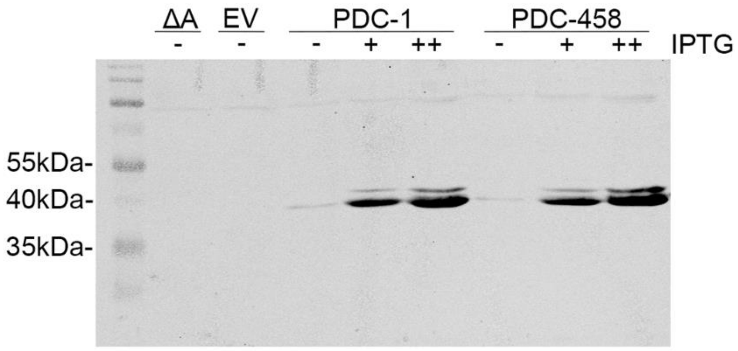

Fig. S3. Representative Western Blot showing the controlled expression of PDC variants PDC-1 and PDC-458.

Entire cell extracts of strain PAΔA expressing either strep-tagged PDC-1 or PDC-458 were used for Western blotting by using specific antibodies against strep-tag. Overnight bacterial cultures were induced with IPTG 10 µM (+), 20 µM (++), or grown without IPTG (-). Cell extracts of PAΔA transformed with the empty vector (EV) pMBLe or with no vector at all (ΔA) were used as negative controls.
