# Supplementary material for: Longitudinal Evolution of the Pseudomonas-Derived Cephalosporinase (PDC) Structure and Activity in a Cystic Fibrosis Patient Treated with β-Lactams
Source: mBio. 2022 Sep 8;13(5):e01663-22. doi: 10.1128/mbio.01663-22 (PMC9600753; doi:10.1128/mbio.01663-22)
Supplement: FIG S4 [file mbio.01663-22-s0004.pdf]

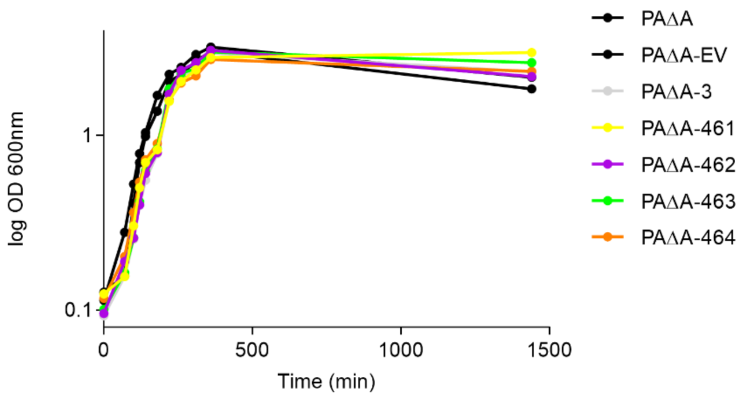

Fig. S4. Growth curves of PAΔA expressing each of PDC variants.

Strains were growth in LB broth supplemented with IPTG 10 μM at 37°C for 16hs. Experiments were performed in duplicates and mean values are shown. No significant differences were observed therefore error bars are omitted for clarity.
