# Supplementary material for: Longitudinal Evolution of the Pseudomonas-Derived Cephalosporinase (PDC) Structure and Activity in a Cystic Fibrosis Patient Treated with β-Lactams
Source: mBio. 2022 Sep 8;13(5):e01663-22. doi: 10.1128/mbio.01663-22 (PMC9600753; doi:10.1128/mbio.01663-22)
Supplement: TEXT S1 [file mbio.01663-22-s0008.docx]

**Supplemental Material for**

**Longitudinal evolution of the Pseudomonas Derived Cephalosporinase (PDC) structure and activity in Cystic Fibrosis patients treated with β-lactams**

Claudia A. Colque^1,2^, Pablo E. Tomatis^3,4¶^, Andrea G. Albarracín Orio^1,2,5¶^, Gina Dotta^3^, Diego M. Moreno^4,6^, Laura G. Hedemann^1,2^, Rachel A. Hickman^7,8^, Lea M. Sommer^7,8^, Sofía Feliziani^1,2^, Alejandro J. Moyano^1,2^, Robert A. Bonomo^9,10^, Helle K. Johansen^7,8,11^, Søren Molin^8^, Alejandro J. Vila^3,4*^, Andrea M. Smania^1,2*^

^1^Universidad Nacional de Córdoba, Facultad de Ciencias Químicas, Departamento de Química Biológica Ranwel Caputto, Córdoba, Argentina.

^2^CONICET, Universidad Nacional de Córdoba, Centro de Investigaciones en Química Biológica de Córdoba (CIQUIBIC), Córdoba, Argentina.

^3^ Instituto de Biología Molecular y Celular de Rosario (IBR), CONICET, Universidad Nacional de Rosario, Rosario, Argentina.

^4^ Facultad de Ciencias Bioquímicas y Farmacéuticas, Universidad Nacional de Rosario, Argentina.

^5^IRNASUS, Universidad Católica de Córdoba, CONICET, Facultad de Ciencias Agropecuarias, Córdoba, Argentina

^6^IQUIR, Instituto de Química de Rosario, CONICET-Universidad Nacional de Rosario, Rosario, Argentina

^7^Department of Clinical Microbiology, Rigshospitalet, Copenhagen, Denmark

^8^Novo Nordisk Foundation Centre for Biosustainability, Technical University of Denmark, Lyngby, Denmark

^9^Department of Molecular Biology and Microbiology, Case Western Reserve University, Cleveland, Ohio, USA

^10^Research Service, Louis Stokes Cleveland Department of Veterans Affairs, Cleveland, Ohio, USA

^11^Department of Clinical Medicine, University of Copenhagen, Copenhagen, Denmark

^¶^These authors contributed equally to this work.

*Correspondence to:

Andrea M. Smania, Centro de Investigaciones en Química Biológica de Córdoba (CIQUIBIC-CONICET), Universidad Nacional de Córdoba, X5000HUA, Córdoba, Argentina, e-mail: asmania@unc.edu.ar.

Alejandro J. Vila, Universidad Nacional de Rosario, Instituto de Biología Molecular y Celular de Rosario (IBR-CONICET), S2000EZP, Rosario, Argentina, e-mail: vila@ibr-conicet.gov.ar

This file includes:

Supplemental Materials and Methods

Supplemental References

**Supplemental Materials and Methods**

**Sequence analysis of** *bla*PDC **gene in *P. aeruginosa* CFD isolates**

The *bla*PDC gene was amplified by PCR directly from bacterial colonies with primers *bla*PDC_FOR and *bla*PDC_REV (Table S5, ResearchGate DOI: 10.13140/RG.2.2.33976.83205). PCR amplifications were performed with the following conditions: 5min at 95°C, 30 cycles of 1 min at 95°C, 50 sec at 52°C, 2 min at 72°C, and a final extension of 10 min at 72°C. PCR products were cleaned with a Silica Purification Kit (Thermo Scientific), and sequenced directly using the same PCR primers (DNA Sequencing Facility, Univ. of Chicago, IL, USA). To score mutations within the gene, *bla*PDC sequences were compared with the corresponding gene sequence of the reference strain PAO1 (www.pseudomonas.com) using the CLC Genomics Workbench 2210.1.1.

**Phylogeny reconstruction of PDC variants.**

Evolutionary analyses were conducted in MEGA11 ([1](#_ENREF_1), [2](#_ENREF_2)). Amino acid sequences of the 12 PDC variants (and the PDC-3) found during the period studied were aligned using MUSCLE software with 16 iterations. Then, evolutionary history was inferred by using the Maximum Likelihood method and JTT matrix-based model ([3](#_ENREF_3)). The tree with the highest log likelihood (-1267.28) is shown. The tree is drawn to scale, with branch lengths measured in the number of substitutions per site. Tree was rooted on PDC-3 variant (the ancestor, as we know). There were a total of 397 positions in the final dataset.

## DNA extraction from sputum samples

For whole gene sequence analysis, genomic DNA was extracted directly from single sputum samples. For this purpose, sputum samples were thaw on ice, and 500 µL of sputum was treated with 30 µL (1M) Tris (2-carboxyethyl) phosphine, 10 µ L proteinase K (20mg/mL) (Thermo Scientific) and 1 mL of DNA shield (Zymo Research) and vortexed for 30 sec. Samples were then added to 2 mL impact resistant screw-top tubes with 300 µL zirconia/glassbeads with a diameter of 0.1 mm (Carl Roth International) and vortexed on a secure horizontal holder at maximum speed for 5 min. Genomic DNA was extracted from the supernatant by ZR-Duet RNA/DNA mini-prep kit (Zymo Research) according to manufacturer’s protocol.

## PCR amplification of *bla*PDC gene from whole sputum samples

## 1.5µl of genomic DNA was used as template for gene amplification using Phusion High-Fidelity PCR master mix (ThermoFischer Scientific) with primers *bla*PDC_FOR and *bla*PDC_REV (Table S5, ResearchGate DOI: 10.13140/RG.2.2.33976.83205). The PCR amplification was performed as follows: 3 min at 98°C, 30 cycles of 10 sec at 98°C, 30 sec at 58°C, 2 min at 72°C and final extension of 5 min at 72°C. To validate correct amplification, amplicons were inspected by gel electrophoresis with GelRed (Biotium).

## Library preparation

PCR product was cleaned with 1.8x AMPure beads (Agencourt®) and 15-30 ng of DNA was used as input for library preparation using KAPPA Hyper Plus kit (Kapa Biosystems) and barcoded using HT-Truseq dual-index adapter kit (Illumina Inc). Library was measured for DNA concentration with Qubit dsDNA HS Assay kit on the Qubit Fluorometer (ThermoFischer Scientific Inc 2015). The average size of the library was measured on the Bioanalyzer with DNA 1000 chip (Agilent Technologies). The sequencing library was sequenced on the MiSeq platform using the MiSeq V2 2x150bp read length kit (Illumina Inc).

## Sequence analysis

Raw sequencing data were processed and trimmed to remove low quality reads. Trimmed reads were aligned to the gene sequence of the PAO1 reference strain (www.pseudomonas.com) by using the CLC Genomics Workbench 10.1.1 (Qiagen) and then both, forward and reverse files, were concatenated into a single file. SNPs were called by using the Low Frequency Variant detector package of CLC Workbench setting the following parameters: min frequency of the variant in the population of 2%, min count of 10 reads supporting the nucleotide position, and min count of 2 reads supporting the variation. From the output file, the only entries considered were the ones with an average quality above 25 and a forward/reverse balance around 0.5. Sequence variants were classified as synonymous (S) or nonsynonymous (NS) and the frequency (%) in the population was scored.

**Construction of *P. aeruginosa* Δ***bla*PDC **deficient strain (PAΔA)**

Strain was constructed by allelic replacement using pKNG101 vector ([4](#_ENREF_4)) and primers *bla*PDC_FOR_Up, *bla*PDC_REV_Up, *bla*PDC_FOR_Down and *bla*PDC_REV_Down (Table S5, ResearchGate DOI: 10.13140/RG.2.2.33976.83205). Briefly, amplicons of 467 and 260 bp from the outside regions of *bla*PDC generated with latter primers, were blunt-ended and then ligated to give a 727 bp fragment which was clone into ApaI and SpeI sites of pKNG101 vector. Conjugation experiments were performed by biparental mating of *E. coli* SM10 pKNG:727 and PAO1. Positive clones were selected by the ability to grow on sucrose 25% and sensibility to streptomycin 50 µg/mL based on the genetic features of pKNG vector. Phenotypic analysis to confirm the deletion of *bla*PDCin the mutant was analyzed by growth inhibition on cefoxitin disks (FOX30µg Britania).

**Construction of *P. aeruginosa*** *bla*PDC**-*lacZ* strain (PAΔA-*lacZ*)**

The PAΔA*-lacZ* strain was constructed by transformation of PAΔA strain with the pUC18-mini Tn7T and pTNS1 plasmids (Table S5, ResearchGate DOI: 10.13140/RG.2.2.33976.83205) by insertion of the β-galactosidase gene at the single attTn7 site downstream of the *glmS* gene in the *P. aeruginosa* genome 15 ([5](#_ENREF_5)). In order to obtain an unmarked PAΔA*-lacZ* strain, the gentamicin cassette, present in the mini Tn7 transposon, was removed by transforming with pFLP2 vector (Table S5, ResearchGate DOI: 10.13140/RG.2.2.33976.83205). Positive clones were selected for the ability to grow on sucrose 10% and sensibility to gentamicin 40 µg/mL and carbenicillin 200 µg/mL. Expression of β-galactosidase (blue colonies) was visualized by the addition of 100 µg/mL X-gal.

**Cloning of** *bla*PDC **allelic variants**

*bla*PDC allelic variants obtained from the CFD collection were cloned into pMBLe ([6](#_ENREF_6)). For this purpose, the complete gene sequence (including native peptide leader) of *bla*PDC was amplified by PCR, from bacterial colonies, with primers *bla*PDC_FOR_NdeI and *bla*PDC_REV_HindIII (for susceptibility testing) and with *bla*PDC_FOR_NdeI and *bla*PDC_REV_HindIII_ST (for Western Blot analyses) (Table S5; ResearchGate DOI: 10.13140/RG.2.2.33976.83205), cloned into NdeI and HindIII sites in pMBLe and transformed into *Escherichia coli* DH5α chemically competent by CaCl2. Transformants were selected on LB agar supplemented with 10 µg/mL of gentamicin. After sequencing step to ensure that no mutation was introduced during PCR amplification, the resulting pMBLe plasmids (Table S5, ResearchGate DOI: 10.13140/RG.2.2.33976.83205) were transferred by electroporation (Bio-Rad MicroPulser) into the PA PAΔA knockout mutant. Transformants were selected on gentamicin 40 µg/mL. The expression of *bla*PDC was induced by addition of 10 µM IPTG verifying that the PAΔA MIC transformed with pMBLe expressing PDC-1 was comparable to that of PAO1 (Table S4). We also verified that the C-terminus Strep tag (added to the cloning for Western Blot analyses purposes), did not affect the ability of PDC to confer resistance.

## PDC expression levels in pMBLe

The *bla*PDC expression levels from pMBLe induced with IPTG were evaluated by Western Blot assays. For this purpose, *bla*PDC allelic variants were labeled with a C-terminal Strep-tag. PAΔA strain was transformed with the pMBLe carrying the different variants, bacteria were grown ON in LB in the presence of 0, 10 and 25µM of IPTG supplemented with 40 µg/ml of gentamicin. Then, 1 mL of each culture was pelleted and resuspended in 20 mM Tris-HCl (pH 7.4), 0.5 M NaCl, 15% glycerol, 1 mM phenylmethylsulfonylfluoride and 1mM benzamidine protease inhibitors. 25 µg of total proteins (measure with Bradford) was separated through sodium dodecyl sulfate (SDS)-polyacrylamide gel electrophoresis (PAGE) 10%, then the proteins were transferred to nitrocellulose membranes (0.22 µm, Sigma) for 1 hour at 300 mA. The blots were blocked for one hour in a 5% milk in phosphate-buffered saline (PBS) solution at room temperature. Incubation with primary antibody (mouse anti-Strep II monoclonal, IBA) was added at 1/10,000 overnight at 4˚C in 5% milk/PBS, then washings were performed with PBS/Tween 20, and the secondary antibody (IR-Dye 800 anti-mouse, LI-COR Bioscience) was added at a 1:10,000 dilution for 1 hour in 5% milk/PBS. Membranes were scanned on the Odyssey infrared imager instrument (LI-COR Bioscience).

## Drug susceptibility testing

MIC determinations were performed by broth dilution method according to CLSI guidelines ([7](#_ENREF_7)). The β-lactam antibiotics tested (breakpoints shown as ≤susceptible/≥resistant) were: ceftazidime (8/32 µg/mL), cefepime (8/32 µg/mL), ceftolozane (4/16 µg/mL), ceftolozane/tazobactam (4-4/16-4 µg/mL), piperacillin (16/128 µg/mL), piperacillin/tazobactam (16-4/128-4 µg/mL), aztreonam (8/32 µg/mL), imipenem (2/8 µg/mL) and meropenem (2/8 µg/mL). *P. aeruginosa* ATCC 27853 was used as control strain.

## Growth curves of PAΔA expressing PDCs cloned into pMBLe

## Isolates were re-grown from frozen in LB plates from which 1 colony was inoculated into 3mL tube for 16hs with shaking. From there, cultures were standardized to an OD600nm of 0.01 and incubated in 15mL flasks for 20 hours at 37 °C with OD600nm measurements every 30 minutes on a spectrophotometer. Flasks were constantly shaken at 150 rpm. Growth curves were measured in duplicated and two independent experiments and the average of OD was plotted as the log 10 versus the time of assessment in minutes.

## Competition experiments

Competitive fitness of *bla*PDC variants was determined by direct competition of each variant PDC-461, PDC-462, PDC-463 and PDC-464 with their ancestral *bla*PDC PDC-3 as well as among variants. For this purpose, PA∆A and PA∆A-*lacZ* strains were transformed with pMBLe-PDC-3, pMBLe-PDC-461, pMBLe-PDC-462, pMBLe-PDC-463 and pMBLe-PDC-464 plasmids to obtain PA∆A-3, PA∆A-461, PA∆A-462, PA∆A-463 and PA∆A-464 strains. For competition experiments, co-cultures at a ratio of 1:1 were inoculated to a final OD of 0.1 and grown in LB medium supplemented with 40 µg/mL gentamicin, 10 µM IPTG, and the presence or absence of different sub_MIC concentrations of ceftazidime or aztreonam for 24 h at 37˚C with shaking. The concentration of antibiotic for a specific competition was defined according to the MIC of the lesser advantage variant. For ceftazidime the concentrations used were as follows: 4xMIC of PDC-3 (16 µg/mL) for competitions relative to PA∆A-3; 1/2xMIC of PDC-464 (32 µg/mL) for competitions relative to PA∆A-464; 1/2xMIC of PDC-463 (32 µg/mL) and 1/4MIC of PDC-462 (32 µg/mL) when competed each other, considering for the latter that 32µg/mL would be enough to differ between PA∆A-462 and PA∆A-461 (MICs of 128 µg/mL). For aztreonam the concentrations used for each experiment were: a 4xMIC of PDC-3 (16 µg/mL) for competitions relative to PA∆A-3; 1/2MIC of PDC-464 (8 µg/mL) for competitions relative to PA∆A-464; 1/2MIC of PDC-461 (16 µg/mL) for competitions relative to PA∆A-461 and 1/2MIC of PDC-462 (32 µg/mL) when competed against PA∆A-462. Afterwards, 100 to 300 cells from the final culture were plated on LB agar plates supplemented with 100 µg/mL X-Gal and grown for 16 h at 37˚C. Blue-white colony screening was used to determine the proportion of *lacZ+* and *lacZ-*, and thus of each variant in the population after competition. To calculate fitness (S) the following equation was used:

𝑆 = ln (*𝛿𝑓*/*𝛿𝑖*) a − ln (*𝛿𝑓*/*𝛿𝑖*) b

Where and *𝛿𝑓* are the number of CFU/mL of initial and final co-cultures; *a* and *b* represent the two competing strains. When S=0, both strains compete the same, and when S˃0 meaning that strain *a* out-competes strain *b*. Two independent experiments, each with three replicates, were assessed for each competition. Two-way analysis of variance (ANOVA) followed by Tukey’s Multiple Comparisons Test of relative fitness values were performed using GraphPad Prism 7.0 Software. Statistically significant differences (P < 0.05) were recorded.

## Expression and purification of PDC proteins

Each *bla*PDC gene encoding mature versions, residues 27 to 397 of the full-length protein, were PCR amplified with primers *bla*PDC_FOR_Mature and *bla*PDC_REV_HindIII (Table S5, ResearchGate DOI: 10.13140/RG.2.2.33976.83205). Then, mature versions were cloned into NdeI and HindIII sites as N-terminal fusion to a 6xHis-tag in the expression vector pET28bTEV ([6](#_ENREF_6)), and transformed into *E. coli* DH5α. Transformants were selected in LB agar plates supplemented with 25 µg/mL of kanamycin. Each pET-PDC plasmid was isolated, verified by PCR and sequencing, and then used to transform *E. coli* BL21 (DE3) strain. For PDC overexpression the strain (BL21::pET-PDC variant) was grown with 25 µg/mL of kanamycin at 37 °C in one liter of LB medium until it reached OD600nm of 0.6, when protein expression was induced by addition of 0.5 mM IPTG, following an incubation with agitation at 20˚C for 20 h. Cell were harvested by centrifugation, resuspended in 25 mL of 50 mM Tris and 200 mM NaCl (Buffer A, pH 8.0) supplemented with DNAse (10 μg/ml) and 50 mM MgCl2. Then, cells were disrupted by sonication (5 times at 40% for 30s and 5 min pause). Afterwards, lysed cells were centrifuged at room temperature or 1 h at 16000 g to remove insoluble materials. Supernatant was loaded at 2 mL/min on a 5 mL HisTrap HP column previously equilibrated with buffer A at 4˚C. Bounded protein to the Ni-Sepharose resin was eluted with a lineal gradient of Buffer A + 500mM Imidazol (pH 8.0). Active fractions were pooled and concentrated using Amicon Ultra 10-15 K down to 5 mL and then subjected to dialysis, for elimination of Imidazol, with 25 mm cellulose membrane against Buffer A at 4 ˚C for 16 h. His-PDC was then digested with His-tagged TEV protease in 30:1 ratio for 2 h at room temperature. PDC protein was then loaded on the HisTrap column and eluted with Buffer A. Purified β-lactamases were concentrated by ultrafiltration using Amicon Ultra 10-15 K to a final concentration of 10 to 30 mg/mL and store at -20˚C. PDC mature protein concentrations were determined from the absorbance at 280 nm using a molar absorption coefficient ε280 of 55,800 M^−1^ cm^−1^ (calculated using Expasy ProtParam, available at http://web.expasy.org/protparam/). All final protein preparations have a purity > 95%, as determined by SDS-PAGE.

## Steady-state kinetic measurements

Purified β-lactamases PDC-3, PDC-461, PDC-462 and PDC-463 were used to determine kinetic parameters using ceftazidime (ΔεM260nm = 9,000 M^-1^ cm^-1^), piperacillin (235nm = 820 M^-1^ cm^-1^) and imipenem (300nm = 9,000 M^-1^ cm^-1^), and ceftolozane (ΔεM263nm = 9,300 M^-1^ cm^-1^) as substrates ([8](#_ENREF_8), [9](#_ENREF_9)). The initial reaction rates at different substrate concentrations were analyze with a Jasco V-670 spectrophotometer at 30˚C in 10 mM phosphate buffer (pH 7.0) in a 0.1cm or 1cm cuvette when appropriate. Spontaneous hydrolysis of antibiotics was monitored by following the absorbance changes of each substrate in the reaction buffer without enzymes. Dependences of initial rates on substrate concentration were analyzed by a nonlinear least squares fit of the data with Michaelis-Menten equation using GraphPad Prism 7.0 in order to determine KM and kcat values. Reported kinetic parameters correspond to averages from at least two determinations with independent protein samples.

## Molecular modeling

**(i) Initial structures.** The initial structure of PDC-3, was built *in-silico* replacing Thr by Ala in position 79 from the crystallographic structure of *Pseudomonas aeruginosa* class C beta-lactamase PDC-1 obtained from the Protein Data Bank entry 4OOY ([10](#_ENREF_10)). The mutants PDC-461, PDC-462 and PDC-463 were constructed replacing the corresponding amino acids.

## (ii) Classical molecular dynamic simulations

MD simulations were performed starting from initial structures built *in-silico* as described above. Each protein was immersed in a truncated octahedral periodic box with a minimum solute-wall distance of 8 Å, filled with explicit TIP3P water molecules ([11](#_ENREF_11)) using the AMBER16 leap module ([12](#_ENREF_12)). Molecular dynamic simulations were performed with the AMBER16 package ([12](#_ENREF_12)), using the ff14SB ([13](#_ENREF_13)) force field. Particle-mesh Ewald (PME) was implemented for long range interactions with a cutoff distance of 12 Å ([14](#_ENREF_14)). Temperature and pressure were regulated with the Berendsen thermostat and barostat, as implemented in the AMBER16 ([12](#_ENREF_12)), using a time constant of 2 ps ([15](#_ENREF_15)). All bonds involving hydrogen were fixed using the SHAKE algorithm ([16](#_ENREF_16)). Each initial system was minimized using a multistep protocol, then heated from 0 to 300 K, and finally a short simulation at constant temperature of 300 K, under constant pressure of 1 bar, was performed to allow the systems to reach proper density. These equilibrated structures were the starting point for 200 ns of MD simulations at 300 K in the NVT ensemble. This protocol was used previously ([17-19](#_ENREF_17)). To analyze the MD simulations, different parameters (Root Mean Square Deviation, Root Mean Square Fluctuation, distances, etc.) were obtained with the cpptraj module ([20](#_ENREF_20)). Conformational clusterization was performed using the hierarchical agglomerative approach from the cpptraj module of Amber16 ([20](#_ENREF_20)).

## (iii) QM-MM calculations

For hybrid QM–MM calculations, we used Self-Consistent Charge Density Functional Tight Binding (SCC-DFTB) ([21](#_ENREF_21)) to describe the QM region as implemented in Amber16 and the same force field used in the classical MD simulations to describe the MM region ([12](#_ENREF_12), [22](#_ENREF_22)). We extracted representative structures of each variant from the MD simulations and performed an structural alignment of them using the VMD software ([23](#_ENREF_23))with the crystallographic structure of a substrate bound PDC (Protein Data Bank entry 1IEL) ([24](#_ENREF_24)). The C-N bond of the beta-lactam ring of the ceftazidime was rebuilt *in silico* to obtain an initial structure of a protein-ceftazidime complex. The simulation protocol consists of an initial minimization at the molecular mechanic level of each complex structure to accommodate solvent molecules and possible clashes, followed by QM-MM geometry optimization. The QM region consisted of the residue of Ser64 and the ceftazidime. We perform two approaches, one with a geometry optimization without restrictions and another with a distance restraint of 2.2 Å between the oxygen atom of Ser64 and the C atom of the carbonyl group of the ceftazidime. We also performed the QM-MM calculations in two steps, first we applied a distance restraint of 2.2 Å between the oxygen atom of Ser64 and the C atom of the carbonyl group of the ceftazidime to accommodate the substrate in catalytic conformation and then we removed the restraint and a full geometry optimization was done. 28

**Supplemental References**

1. Tamura K, Stecher G, Kumar S. 2021. MEGA11: Molecular Evolutionary Genetics Analysis Version 11. Mol Biol Evol 38:3022-3027.

2. Stecher G, Tamura K, Kumar S. 2020. Molecular Evolutionary Genetics Analysis (MEGA) for macOS. Mol Biol Evol 37:1237-1239.

3. Jones DT, Taylor WR, Thornton JM. 1992. The rapid generation of mutation data matrices from protein sequences. Comput Appl Biosci 8:275-82.

4. Kaniga K, Delor I, Cornelis GR. 1991. A wide-host-range suicide vector for improving reverse genetics in gram-negative bacteria: inactivation of the *blaA* gene of *Yersinia enterocolitica*. Gene 109:137-41.

5. Choi KH, Gaynor JB, White KG, Lopez C, Bosio CM, Karkhoff-Schweizer RR, Schweizer HP. 2005. A Tn7-based broad-range bacterial cloning and expression system. Nat Methods 2:443-8.

6. González LJ, Bahr G, Nakashige TG, Nolan EM, Bonomo RA, Vila AJ. 2016. Membrane anchoring stabilizes and favors secretion of New Delhi metallo-β-lactamase. Nature chemical biology 12:516-522.

7. CLSI. 2019. Performance Standards for Antimicrobial Susceptibility Testing. 29th ed. Wayne, PA: Clinical and Laboratory Standards Institute.

8. Felici A, Amicosante G, Oratore A, Strom R, Ledent P, Joris B, Fanuel L, Frère JM. 1993. An overview of the kinetic parameters of class B beta-lactamases. The Biochemical journal 291 ( Pt 1):151-155.

9. Barnes MD, Taracila MA, Rutter JD, Bethel CR, Galdadas I, Hujer AM, Caselli E, Prati F, Dekker JP, Papp-Wallace KM, Haider S, Bonomo RA. 2018. Deciphering the evolution of cephalosporin resistance to ceftolozane-tazobactam in *Pseudomonas aeruginosa*. mBio 9:e02085-18.

10. Lahiri SD, Johnstone MR, Ross PL, McLaughlin RE, Olivier NB, Alm RA. 2014. Avibactam and class C beta-lactamases: mechanism of inhibition, conservation of the binding pocket, and implications for resistance. Antimicrob Agents Chemother 58:5704-13.

11. Jorgensen WL, Chandrasekhar J, Madura JD, Impey RW, Klein ML. 1983. Comparison of simple potential functions for simulating liquid water. The Journal of Chemical Physics 79:926-935.

12. D.A. Case, R.M. Betz, D.S. Cerutti, T.E. Cheatham, III, T.A. Darden, R.E. Duke, T.J. Giese, H. Gohlke, A.W. Goetz, N. Homeyer, S. Izadi, P. Janowski, J. Kaus, A. Kovalenko, T.S. Lee, S. LeGrand, P. Li, C. Lin, T. Luchko, R. Luo, B. Madej, D. Mermelstein, K.M. Merz, G. Monard, H. Nguyen, H.T. Nguyen, I. Omelyan, A. Onufriev, D.R. Roe, A. Roitberg, C. Sagui, C.L. Simmerling, W.M. Botello-Smith, J. Swails, R.C. Walker, J. Wang, R.M. Wolf, X. Wu, L. Xiao, Kollman PA. 2016. AMBER 2016, University of California, San Francisco,

13. Maier JA, Martinez C, Kasavajhala K, Wickstrom L, Hauser KE, Simmerling C. 2015. ff14SB: Improving the Accuracy of Protein Side Chain and Backbone Parameters from ff99SB. Journal of Chemical Theory and Computation 11:3696-3713.

14. Luty BA, Tironi IG, Gunsteren WFv. 1995. Lattice‐sum methods for calculating electrostatic interactions in molecular simulations. The Journal of Chemical Physics 103:3014-3021.

15. Berendsen HJC, Postma JPM, Gunsteren WFv, DiNola A, Haak JR. 1984. Molecular dynamics with coupling to an external bath. The Journal of Chemical Physics 81:3684-3690.

16. Ryckaert J-P, Ciccotti G, Berendsen HJC. 1977. Numerical integration of the cartesian equations of motion of a system with constraints: molecular dynamics of n-alkanes. Journal of Computational Physics 23:327-341.

17. González LJ, Moreno DM, Bonomo RA, Vila AJ. 2014. Host-Specific Enzyme-Substrate Interactions in SPM-1 Metallo-β-Lactamase Are Modulated by Second Sphere Residues. PLOS Pathogens 10:e1003817.

18. Morán-Barrio J, Lisa M-N, Larrieux N, Drusin SI, Viale AM, Moreno DM, Buschiazzo A, Vila AJ. 2016. Crystal Structure of the Metallo-β-Lactamase GOB in the Periplasmic Dizinc Form Reveals an Unusual Metal Site. Antimicrobial Agents and Chemotherapy 60:6013-6022.

19. González LJ, Stival C, Puzzolo JL, Moreno DM, Vila AJ. 2018. Shaping Substrate Selectivity in a Broad-Spectrum Metallo-β-Lactamase. Antimicrobial Agents and Chemotherapy 62:e02079-17.

20. Roe DR, Cheatham TE. 2013. PTRAJ and CPPTRAJ: Software for Processing and Analysis of Molecular Dynamics Trajectory Data. Journal of Chemical Theory and Computation 9:3084-3095.

21. Gaus M, Cui Q, Elstner M. 2011. DFTB3: Extension of the Self-Consistent-Charge Density-Functional Tight-Binding Method (SCC-DFTB). Journal of Chemical Theory and Computation 7:931-948.

22. Seabra GdM, Walker RC, Elstner M, Case DA, Roitberg AE. 2007. Implementation of the SCC-DFTB Method for Hybrid QM/MM Simulations within the Amber Molecular Dynamics Package. The Journal of Physical Chemistry A 111:5655-5664.

23. Humphrey W, Dalke A, Schulten K. 1996. VMD: Visual molecular dynamics. Journal of Molecular Graphics 14:33-38.

24. Powers RA, Caselli E, Focia PJ, Prati F, Shoichet BK. 2001. Structures of Ceftazidime and Its Transition-State Analogue in Complex with AmpC β-Lactamase:  Implications for Resistance Mutations and Inhibitor Design. Biochemistry 40:9207-9214.

25. Rodríguez-Martínez J-M, Poirel L, Nordmann P. 2009. Extended-Spectrum Cephalosporinases in *Pseudomonas aeruginosa*. Antimicrobial Agents and Chemotherapy 53:1766-1771.

26. Berrazeg M, Jeannot K, Ntsogo Enguene VY, Broutin I, Loeffert S, Fournier D, Plesiat P. 2015. Mutations in beta-lactamase AmpC increase resistance of *Pseudomonas aeruginosa* isolates to antipseudomonal cephalosporins. Antimicrob Agents Chemother 59:6248-55.

27. Marvig RL, Johansen HK, Molin S, Jelsbak L. 2013. Genome analysis of a transmissible lineage of *Pseudomonas aeruginosa* reveals pathoadaptive mutations and distinct evolutionary paths of hypermutators. PLOS Genetics 9:e1003741.

28. Haidar G, Philips NJ, Shields RK, Snyder D, Cheng S, Potoski BA, Doi Y, Hao B, Press EG, Cooper VS, Clancy CJ, Nguyen MH. 2017. Ceftolozane-Tazobactam for the Treatment of Multidrug-Resistant *Pseudomonas aeruginosa* Infections: Clinical Effectiveness and Evolution of Resistance. Clinical Infectious Diseases 65:110-120.

29. López-Causapé C, Sommer LM, Cabot G, Rubio R, Ocampo-Sosa AA, Johansen HK, Figuerola J, Cantón R, Kidd TJ, Molin S, Oliver A. 2017. Evolution of the *Pseudomonas aeruginosa* mutational resistome in an international cystic fibrosis clone. Scientific reports 7:5555-5555.

30. Oliver A. 2020. Antibiotic Resistance and Pathogenicity of Bacterial Infections Group - IdISBa

31. Holloway BW. 1955. Genetic Recombination in *Pseudomonas aeruginosa*. Microbiology 13:572-581.

32. Miller VL, Mekalanos JJ. 1988. A novel suicide vector and its use in construction of insertion mutations: osmoregulation of outer membrane proteins and virulence determinants in *Vibrio cholerae* requires toxR. Journal of Bacteriology 170:2575-2583.

33. Simon R, Priefer U, Pühler A. 1983. A Broad Host Range Mobilization System for In Vivo Genetic Engineering: Transposon Mutagenesis in Gram Negative Bacteria. Bio/Technology 1:784-791.

34. Choi K-H, Schweizer HP. 2006. mini-Tn7 insertion in bacteria with single attTn7 sites: example *Pseudomonas aeruginosa*. Nature Protocols 1:153-161.

35. Hoang TT, Karkhoff-Schweizer RR, Kutchma AJ, Schweizer HP. 1998. A broad-host-range Flp-FRT recombination system for site-specific excision of chromosomally-located DNA sequences: application for isolation of unmarked *Pseudomonas aeruginosa* mutants. Gene 212:77-86.

36. Tomalka AG, Zmina SE, Stopford CM, Rietsch A. 2013. Dimerization of the *Pseudomonas aeruginosa* translocator chaperone PcrH is required for stability, not function. Journal of bacteriology 195:4836-4843.
