# Supplementary material for: Longitudinal Evolution of the Pseudomonas-Derived Cephalosporinase (PDC) Structure and Activity in a Cystic Fibrosis Patient Treated with β-Lactams
Source: mBio. 2022 Sep 8;13(5):e01663-22. doi: 10.1128/mbio.01663-22 (PMC9600753; doi:10.1128/mbio.01663-22)
Supplement: FIG S5 [file mbio.01663-22-s0005.pdf]

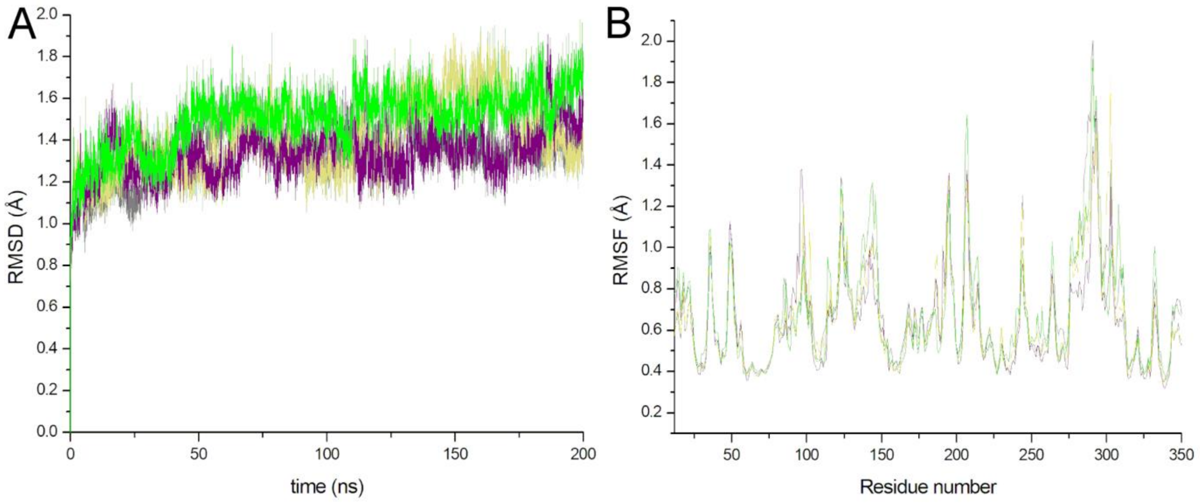

Fig. S5. RMSD (A) and RMSF (B) values calculated during the MD simulations of the different proteins studied: PDC-3 (gray), PDC-461 (yellow), PDC-462 (purple) and PDC-463 (green).
